# Removal of Medicaid Prior Authorization Requirements and Buprenorphine Treatment for Opioid Use Disorder

**DOI:** 10.1001/jamahealthforum.2023.3549

**Published:** 2023-10-20

**Authors:** Paul J. Christine, Marc R. Larochelle, Lewei (Allison) Lin, Jonathon McBride, Renuka Tipirneni

**Affiliations:** 1 Section of General Internal Medicine, Department of Medicine, University of Colorado School of Medicine, Aurora; 2 Section of General Internal Medicine, Department of Medicine, Boston Medical Center, Boston, Massachusetts; 3 Section of General Internal Medicine, Department of Medicine, Boston University Chobanian and Avedisian School of Medicine, Boston, Massachusetts; 4 VA Center for Clinical Management Research, VA Ann Arbor Healthcare System, Ann Arbor, Michigan; 5 Addiction Center, Department of Psychiatry, University of Michigan Medical School, Ann Arbor; 6 Department of Anesthesiology, University of Michigan Medical School, Ann Arbor; 7 Division of General Medicine, Department of Internal Medicine, University of Michigan Medical School, Ann Arbor

## Abstract

**Importance:**

Buprenorphine treatment for opioid use disorder (OUD) is associated with decreased morbidity and mortality. Despite its effectiveness, buprenorphine uptake has been limited relative to the burden of OUD. Prior authorization (PA) policies may present a barrier to treatment, though research is limited, particularly in Medicaid populations.

**Objective:**

To assess whether removal of Medicaid PAs for buprenorphine to treat OUD is associated with changes in buprenorphine prescriptions for Medicaid enrollees.

**Design, Setting, and Participants:**

This state-level, serial cross-sectional study used quarterly data from 2015 through the first quarter (January-March) of 2019 to compare buprenorphine prescriptions in states that did and did not remove Medicaid PAs. Analyses were conducted between June 10, 2021, and August 15, 2023. The study included 23 states with active Medicaid PAs for buprenorphine in 2015 that required similar PA policies in fee-for-service and managed care plans and had at least 2 quarters of pre- and postperiod buprenorphine prescribing data.

**Exposures:**

Removal of Medicaid PA for at least 1 formulation of buprenorphine for OUD.

**Main Outcomes and Measures:**

The main outcome was number of quarterly buprenorphine prescriptions per 1000 Medicaid enrollees.

**Results:**

Between 2015 and the first quarter of 2019, 6 states in the sample removed Medicaid PAs for at least 1 formulation of buprenorphine and had at least 2 quarters of pre- and postpolicy change data. Seventeen states maintained buprenorphine PAs throughout the study period. At baseline, relative to states that repealed PAs, states that maintained PAs had lower buprenorphine prescribing per 1000 Medicaid enrollees (median, 6.6 [IQR, 2.6-13.9] vs 24.1 [IQR, 8.7-27.5] prescriptions) and lower Medicaid managed care penetration (median, 38.5% [IQR, 0.0%-74.1%] vs 79.5% [IQR, 78.1%-83.5%] of enrollees) but similar opioid overdose rates and X-waivered buprenorphine clinicians per 100 000 population. In fully adjusted difference-in-differences models, removal of Medicaid PAs for buprenorphine was not associated with buprenorphine prescribing (1.4% decrease; 95% CI, −31.2% to 41.4%). For states with below-median baseline buprenorphine prescribing, PA removal was associated with increased buprenorphine prescriptions per 1000 Medicaid enrollees (40.1%; 95% CI, 0.6% to 95.1%), while states with above-median prescribing showed no change (−20.7%; 95% CI, −41.0% to 6.6%).

**Conclusions and Relevance:**

In this serial cross-sectional study of Medicaid PA policies for buprenorphine for OUD, removal of PAs was not associated with overall changes in buprenorphine prescribing among Medicaid enrollees. Given the ongoing burden of opioid overdoses, continued multipronged efforts are needed to remove barriers to buprenorphine care and increase availability of this lifesaving treatment.

## Key Points

**Question:**

Is there an association between removal of Medicaid prior authorization (PA) for buprenorphine to treat opioid use disorder (OUD) and buprenorphine prescriptions for Medicaid enrollees?

**Findings:**

In this serial cross-sectional study of 23 states from 2015 to 2019, the removal of Medicaid PA for buprenorphine to treat OUD was not associated with substantial overall changes in buprenorphine prescriptions. Prior authorization removal was associated with increased buprenorphine prescribing in states with low baseline buprenorphine prescribing but not in states with higher baseline prescribing.

**Meaning:**

These findings suggest that removing PAs for buprenorphine to treat OUD alone may not substantially increase buprenorphine prescribing among Medicaid enrollees.

## Teaser

This cross-sectional study assesses the association of removing prior authorization for buprenorphine to treat opioid use disorder with prescribing practices for Medicaid enrollees.

## Introduction

State Medicaid programs use a variety of utilization management policies, including prior authorization (PA), to ensure safe delivery of care while containing costs and minimizing resource misuse. Several such policies have been directed at buprenorphine, a highly effective medication used to treat opioid use disorder (OUD).^1^ Despite reducing morbidity and mortality among patients with OUD, buprenorphine treatment uptake has been limited relative to the burden of OUD and overdose deaths.^2^

One potential contributor to the limited uptake of buprenorphine treatment is PA requirements. Prior authorizations for buprenorphine are a frequently cited barrier to care, with both clinicians and treatment advocates calling for their removal as a way to increase treatment.^3,4^ Prior work suggests that state Medicaid programs that require PAs for buprenorphine have fewer addiction treatment facilities that offer buprenorphine.^5^ In the Medicare population, removing PAs for buprenorphine-naloxone (the most common formulation for OUD) was associated with increased prescriptions and decreased OUD-related inpatient admissions and emergency department visits.^6^ The sole study in the Medicaid population that evaluated PA removal focused only on 2 states and found mixed results, showing 1 state with increased buprenorphine prescriptions after PA removal and the other showing no change.^7^ Given that Medicaid is the largest insurer of individuals with OUD, there is a need for additional research evaluating the association of PA policies with buprenorphine prescriptions in this population.^8^

Using serial cross-sectional data on Medicaid PA policies and buprenorphine prescriptions from 2015 to 2019, we sought to examine whether changes in state Medicaid PA requirements were associated with changes in buprenorphine prescriptions over time. We also examined whether PA removal was associated with differential changes in buprenorphine prescribing according to baseline state characteristics, including level of buprenorphine prescribing, Medicaid expansion status, and Medicaid managed care penetration.

## Methods

### Study Population and Analytic Sample

We conducted a retrospective, repeated cross-sectional study at the state level using quarterly data from the first quarter (January-March) of 2015 through the first quarter (January-March) of 2019. We limited our analysis to states with PAs in place at the start of 2015 (n = 48) that required fee-for-service (FFS) and managed care organization (MCO) plans to have similar PA policies for buprenorphine and that had at least 2 quarters of prescription data before and after a PA was removed (n = 25). We also excluded Vermont due to buprenorphine prescribing rates that were considerably higher than all other states and Illinois due to a protracted period when it was the only state that had repealed its PA. Our final sample includes 23 states with 414 state-quarters of data (eFigure 1 and eAppendix in Supplement 1). Based on the information provided, the University of Michigan institutional review board determined that this research was exempt from the need for informed consent due to the use of publicly available and individually unidentifiable data as defined in 45 CFR 46.101(2)(b). The Strengthening the Reporting of Observational Studies in Epidemiology (STROBE) guideline was used to guide reporting of this study.

### PA Policies

Medicaid PA policies were reviewed from January 1 through April 30, 2021, and were obtained from a variety of sources. We performed an extensive search of state registers, archived preferred drug lists, published literature, and news reports to establish the Medicaid PA policy status of each state (eAppendix and eTable 1 in Supplement 1). For each quarter during our study, we categorized states as PA present or PA absent. For states removing PAs during the study period, the date of removal (or effective date, if different) was noted and their category switched to PA absent in the same quarter, hypothesizing that the policy change would have an immediate effect (eFigure 2 in Supplement 1). States were considered to have removed their PA if they made at least 1 formulation of buprenorphine for OUD available without a PA, though they may have maintained PAs for other formulations or for higher doses (eg, >16 mg). For most states, PA policy documentation came from Medicaid FFS plans, though in some states, we were able to find PA policies for MCO plans as well. Because complete PA policy information for Medicaid MCO plans was not available during our study time frame, we restricted our sample to states where PA policies in Medicaid FFS apply to all Medicaid enrollees (eAppendix in Supplement 1).

### Buprenorphine Prescriptions

Buprenorphine prescription counts came from Medicaid State Drug Utilization Data, courtesy of Lisa Clemans-Cope, PhD, at the Urban Institute.^9^ The Medicaid State Drug Utilization Data contain outpatient medication data for all medications covered under the Medicaid Drug Rebate Program.^10^ National Drug Codes were used to identify buprenorphine medications that are approved by the US Food and Drug Administration for treating OUD (eTable 5 in Supplement 1). Our outcome of interest was quarterly buprenorphine prescriptions per 1000 Medicaid enrollees aged 12 years or older calculated using Medicaid state enrollment data.^11^ Data were available through the first quarter of 2019.

### Covariates

We collected data on a variety of state-level characteristics related to OUD burden, sociodemographics, and opioid health policy, including annual opioid overdose rate per 100 000 adults, annual number of clinicians certified to prescribe buprenorphine for OUD (Drug Enforcement Administration X-waivered clinicians) per 100 000 population, annual number of individuals receiving methadone through an opioid treatment program per 100 000 adults, annual percentage of individuals aged 12 years or older living below the federal poverty limit, state Medicaid expansion status, state proportion of individuals with Medicaid covered under comprehensive managed care plans, and the presence of state mandates to use prescription drug monitoring programs (PDMPs) when prescribing opioids (see eAppendix in Supplement 1 for data sources).

### Statistical Analysis

We calculated descriptive statistics for various state characteristics in 2015, comparing states that did and did not remove Medicaid buprenorphine PAs during the study period. To evaluate changes in buprenorphine prescriptions per 1000 Medicaid enrollees, we used an event-study framework to compare the number of prescriptions among states that removed buprenorphine PAs vs states that did not remove buprenorphine PAs before and after each state’s quarter of policy change. Because the distribution of buprenorphine prescriptions was right skewed, we log transformed the outcome in all models. Effect estimates are provided as the estimated percent change in buprenorphine prescriptions per 1000 Medicaid enrollees. Event study models included an indicator for PA policy (0 if PA present, 1 if PA removed), an indicator for quarter relative to the PA policy change, an interaction term between PA policy and quarter, state fixed effects, year fixed effects, and the following state-level covariates: opioid overdose deaths per 100 000 adults, log number of X-waivered buprenorphine clinicians, number of individuals receiving methadone through an opioid treatment program per 100 000 adults (including quadratic term), percent of individuals older than 12 years living below the federal poverty limit, Medicaid expansion status, proportion of a state’s Medicaid enrollees covered under a comprehensive managed care plan, and state PDMP status. We assessed the preperiod parallel trends assumption using event-study plots, and trends appeared to be parallel. We estimated the association of PA removal with buprenorphine prescriptions using a difference-in-differences approach with 2-way fixed effects. Specifically, we estimated a log-linear regression model at the state-quarter level that included an indicator for time-varying PA policy status, state and year fixed effects, and all of the covariates listed above (eAppendix in Supplement 1). The SEs were clustered at the state level.

We hypothesized a priori that baseline state characteristics may be associated with differential effects of PA removal on buprenorphine prescribing. To assess this association, we conducted difference-in-difference-in-differences (triple-difference) models that interact time-varying PA policy status with baseline state characteristics, including buprenorphine prescribing (above vs below median), Medicaid MCO penetration (above vs below median), and Medicaid expansion status. All triple-difference models controlled for the same covariates as the main models.

We performed a number of sensitivity analyses. First, to assess the sensitivity of our results to restricting our sample to states where PA policies in Medicaid FFS apply to all Medicaid enrollees, we reran our analyses on 2 expanded samples of states that included Illinois and states where PA policies are allowed to differ between FFS and MCO plans (eAppendix and eFigure 4 in Supplement 1). Second, because traditional 2-way fixed-effects models may produce biased estimates when policy changes are staggered in time, we used 2 alternative difference-in-difference estimators and the Bacon decomposition method to assess for such biases (eAppendix in Supplement 1).^12^ We also assessed the sensitivity of our difference-in-differences methods by using a generalized synthetic control model to estimate mean differences in log buprenorphine prescriptions by comparing states that removed PAs with a hypothetical counterfactual synthetic control group (eAppendix in Supplement 1).^13^ Third, to assess the possibility that buprenorphine prescribing takes time to ramp up after PA removal, we reran our main models with 6-month and 12-month lags for PA removal. Finally, because several states had PA policies that were difficult to interpret or involved nuances that could influence the effect of removing the PA, we performed multiple sensitivity analyses that reclassified state PA policies to assess the robustness of our main results. All analyses were conducted between June 10, 2021, and August 15, 2023, using SAS, version 9.4 (SAS Institute Inc) and R, version 4.1.1 (R Foundation for Statistical Computing) (see eAppendix in Supplement 1 for specific R packages used).

## Results

**Figure 1.**
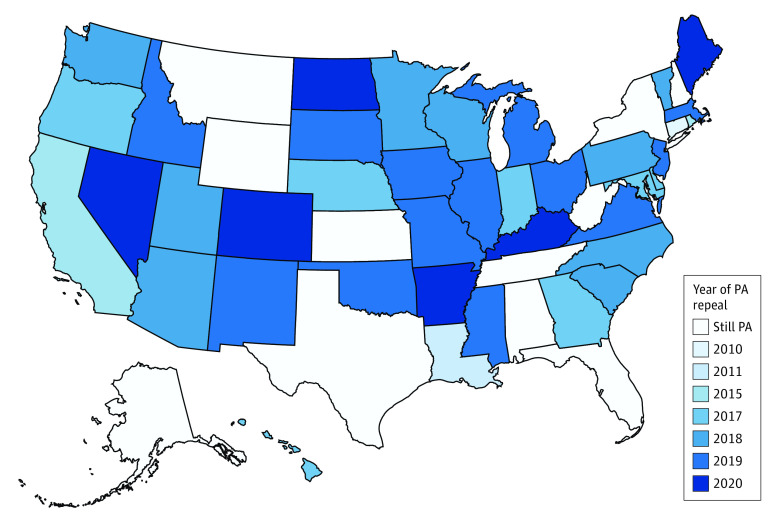
Map of Medicaid Prior Authorization (PA) Status for Buprenorphine as of January 2021

Between 2015 and the first quarter of 2019, 23 states removed Medicaid PAs for at least 1 formulation of buprenorphine for OUD, of which 6 had at least 2 quarters of pre- and postpolicy change data and had PA policies that applied to both FFS and MCO plans (Figure 1; eFigure 2 in Supplement 1). Seventeen states maintained their buprenorphine PAs throughout the study period. In general, most states experienced increases in buprenorphine prescribing per 1000 Medicaid enrollees throughout the study period (eFigure 3 in Supplement 1).

Compared with states that removed a buprenorphine PA during the study period, states that maintained buprenorphine PAs differed in several ways at baseline, including a lower number of buprenorphine prescriptions per 1000 Medicaid enrollees (median, 6.6 [IQR, 2.6-13.9] vs 24.1 [IQR, 8.7-27.5]), higher percentage of individuals living below the federal poverty limit (mean [SE], 15.3 [0.7] vs 13.6 [1.1]), lower likelihood of having expanded Medicaid (5 states [29.4%] vs 4 states [66.7%]), fewer Medicaid enrollees covered under MCO plans (median, 38.5% [IQR, 0.0%-74.1%] vs 79.5% [IQR, 78.1%-83.5%]), and fewer individuals receiving methadone through an opioid treatment program per 100 000 adults (median, 88.3 [IQR, 50.5-128.2] vs 191.8 [IQR, 151.3-389.9]) (Table 1). States generally had similar levels of opioid overdose per 100 000 adults, X-waivered buprenorphine clinicians per 100 000 population, and mandated PDMP reviews.

**Table 1.** Baseline Descriptive Characteristics of States by Medicaid Buprenorphine Prior Authorization (PA) Status, First Quarter (January-March) of 2015^a^

State characteristic	States not removing PA (n = 17)	States removing PA (n = 6)
Buprenorphine prescriptions per 1000 Medicaid enrollees, median (IQR)	6.6 (2.6-13.9)	24.1 (8.7-27.5)
Opioid overdose death rate per 100 000 adults, median (IQR)	9.4 (6.1-14.1)	11.6 (10.5-14.1)
No. of X-waivered clinicians per 100 000 population, median (IQR)	1.0 (0.6-2.3)	1.8 (1.1-2.3)
Percentage of individuals aged >12 y living below the federal poverty limit, mean (SE)	15.3 (0.7)	13.6 (1.1)
Expanded Medicaid, No. of states (%)	5 (29.4)	4 (66.7)
Percentage of individuals with Medicaid covered under managed care, median (IQR)	38.5 (0.0-74.1)	79.5 (78.1-83.5)
PDMP review mandate, No. of states (%)	3 (17.7)	1 (16.7)
No. of individuals receiving methadone through an opioid treatment program per 100 000 adults, median (IQR)	88.3 (50.5-128.2)	191.8 (151.3-389.9)

Abbreviation: PDMP, prescription drug monitoring program.

^a^ Authors’ analysis of data from the following sources in 2015: Medicaid State Drug Utilization Data, Centers for Disease Control and Prevention WONDER (Wide-Ranging Online Data for Epidemiologic Research) Multiple Cause of Death Files, Substance Abuse Mental Health Services Administration, National Survey of Substance Abuse Treatment Services, US Census Bureau’s Small Area Income and Poverty Estimates Program, and Kaiser Family Foundation.

**Figure 2.**
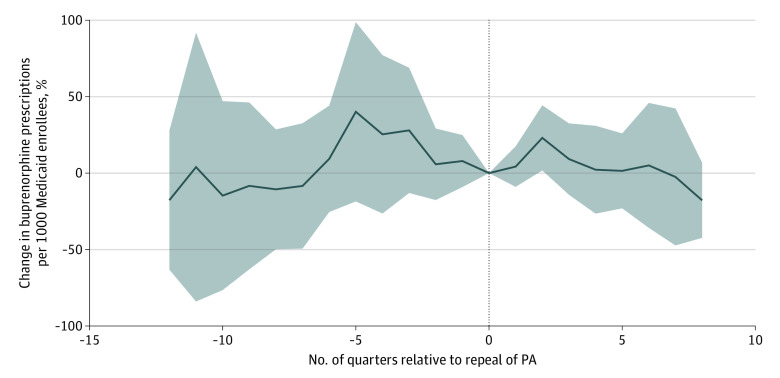
Estimated Change in Buprenorphine Prescriptions per 1000 Medicaid Enrollees Comparing States that Removed Prior Authorization (PA) vs Those That Maintained PAs, 2015-2019 Percent change estimates come from an event study model that controls for state fixed effects, year fixed effects, and the state-level covariates listed in the Statistical Analysis section. The SEs are clustered at the state level. Using a 2-way fixed-effects difference-in-differences model adjusted for all of the state-level covariates, PA removal was not associated with significant changes in buprenorphine prescriptions per 1000 Medicaid enrollees (−1.4% increase; 95% CI, −31.2% to 41.4%). Solid line indicates percent change; shaded area, 95% CI; dotted line, repeal of PAs for buprenorphine.

Figure 2 shows fully adjusted event study estimates of percent change in buprenorphine prescriptions per 1000 Medicaid enrollees, comparing states that did vs did not remove PAs during the study period. The data suggest that the removal of PAs was not associated with consistent changes in buprenorphine prescriptions per 1000 Medicaid enrollees. In the standard 2-way fixed-effects difference-in-differences model controlling for all covariates, removing buprenorphine PAs was not associated with buprenorphine prescribing (−1.4% increase in buprenorphine prescriptions per 1000 Medicare enrollees; 95% CI, −31.2% to 41.4%).

**Figure 3.**
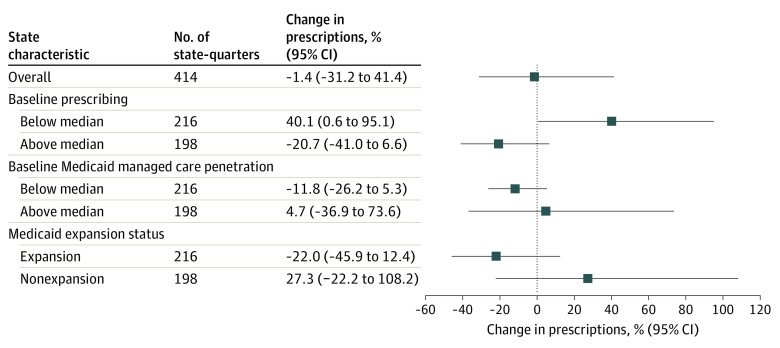
Estimated Change in Buprenorphine Prescriptions per 1000 Medicaid Enrollees Associated With Removal of Prior Authorizations (PAs) Overall and by Categories of Baseline State Characteristics, 2015-2019 The overall model represents a difference-in-differences estimate of percent change in buprenorphine prescriptions per 1000 Medicaid enrollees, comparing states that removed PAs vs those that maintained PAs. Models evaluating heterogeneity by baseline prescribing, Medicaid managed care penetration, and Medicaid expansion status used a difference-in-difference-in-differences design. Models controlled for state fixed effects, year fixed effects, and the state-level covariates listed in the Statistical Analysis section. The SEs are clustered at the state level.

Figure 3 shows fully adjusted triple-difference estimates by baseline state characteristics. Buprenorphine PA removal had different effects for states with below- vs above-median baseline buprenorphine prescribing, with below-median states showing an increase in prescriptions after PA removal that was not observed in above-median states (40.1% increase [95% CI, 0.6% to 95.1%] vs 20.7% decrease [95% CI, −41.0% to 6.6%]). The effect of buprenorphine PA removal did not differ by baseline Medicaid MCO penetration, though estimates were imprecise (11.8% decrease [95% CI, −26.2% to 5.3%] in below-median states vs 4.7% increase [95% CI, −36.9% to 73.6%] in above-median states). For Medicaid expansion status, nonexpansion states showed an increase in buprenorphine prescriptions after PA removal (27.3% increase; 95% CI, −22.2% to 108.2%), while expansion states showed a decrease in prescriptions (22.0% decrease; 95% CI, −45.9% to 12.4%), though estimates were again imprecise.

Sensitivity analyses generally showed that the results are robust to a variety of model specifications. Analyses expanding the sample to include Illinois and states where Medicaid PA policies are allowed to differ between FFS and MCO plans produced qualitatively similar results (Table 2; eFigures 5 and 6 in Supplement 1). Our difference-in-differences estimates largely used appropriate comparisons (eFigure 7 in Supplement 1), and alternative difference-in-differences estimators produced similar estimates (eFigure 8 and eTable 2 in Supplement 1). Effect estimates from generalized synthetic control models were also similar, though imprecise (eFigure 9 and eTable 2 in Supplement 1). Results were generally robust to different policy lags, to reassigning PA policy status for several states, and to the inclusion of Vermont (eTables 3 and 4 in Supplement 1).

**Table 2.** Sensitivity Analyses Using Alternative Samples of States to Estimate the Change in Buprenorphine Prescriptions Associated With Removal of Prior Authorization (PA) Requirements^a^

Model	No. of states	Change in buprenorphine prescriptions per 1000 Medicaid enrollees, % (95% CI)
Main sample^b^	23	−1.4 (−31.2 to 41.4)
Main sample + Illinois^c^	24	11.9 (−24.6 to 66.0)
Main sample + Illinois + states where FFS and MCO PA policies are allowed to differ^d^	40	7.5 (−11.6 to 30.8)

Abbreviations: FFS, fee for service; MCO, managed care organization.

^a^ Difference-in-differences estimates from 2-way fixed-effects models. All models control for state fixed effects, year fixed effects, and the state-level covariates listed in the Statistical Analysis section. The SEs are clustered at the state level.

^b^ The main sample includes states where Medicaid FFS PA policies are known to apply to all Medicaid enrollees, including those in Medicaid MCOs. To be included in this restricted sample, a state must meet at least 1 of the following criteria throughout the study period: (1) the state has no Medicaid MCOs (ie, is all FFS), (2) the state MCO pharmacy benefits are carved out to FFS Medicaid, and (3) the state MCO utilization management policies must either follow or be no more restrictive than FFS policies.

^c^ This sample applies the same restriction criteria as the main sample but includes Illinois, which is the only state that contributes to the effect estimates after the ninth quarter in the postperiod.

^d^ This sample includes states in the main sample and Illinois, as well as other states where FFS and MCO plans are allowed to have different PA policies.

## Discussion

Using state-level data from 2015 to 2019 during a time of rapid change in Medicaid buprenorphine PA policies, we observed little evidence that removing PAs was associated with changes in buprenorphine prescriptions among Medicaid enrollees. During the post-PA removal period, states that removed buprenorphine PAs did not experience a differential change in buprenorphine prescriptions per 1000 Medicaid enrollees relative to states maintaining buprenorphine PAs. The lack of association between PA removal and buprenorphine prescriptions generally remained true across various state characteristics, though states with lower baseline buprenorphine prescriptions showed an increase in prescribing. These results indicate that efforts to remove Medicaid buprenorphine PAs alone may not result in meaningful increases in prescriptions.

The uptake of buprenorphine for OUD is influenced by a variety of factors at multiple levels along the cascade of care.^14^ At the policy level, prior regulatory requirements to obtain an X-waiver, inadequate reimbursement, and scope of practice regulations limit buprenorphine adoption by many clinicians.^15,16^ At the clinician level, clinicians cite insufficient training regarding OUD, lack of institutional support, and insurance hurdles such as PAs as common barriers to providing buprenorphine treatment.^17,18,19^ As a result, less than 4% of eligible clinicians prescribe buprenorphine for OUD and typically to only a few patients at a time.^15,20^ For patients, experiences of stigma, preference for more autonomy relative to methadone, and desire for a more patient-centered experience also influence the desirability of buprenorphine treatment.^21,22^

Notwithstanding the multilevel factors at play in buprenorphine treatment, PAs have garnered specific attention as a barrier to addiction care.^3,4^ Critics of buprenorphine PAs point out that many PA requirements are not supported by evidence, including mandatory counseling, dose limits, and predefined tapering schedules.^4,23,24^ These unnecessarily restrictive requirements run contrary to growing evidence that low-barrier, on-demand addiction care can increase treatment engagement and retention, including in historically marginalized populations.^25,26,27,28^

While fairly limited to date, research on the outcomes associated with buprenorphine PAs has been mixed. In the most comprehensive study to date using Medicare claims data, Mark et al^6^ found that removal of buprenorphine-naloxone PAs in Medicare Part D plans was associated with an increase of 17.9 prescriptions filled per plan per year, equivalent to approximately a doubling of the number of prescriptions. They also found that PA removal for buprenorphine-naloxone was associated with small decreases in substance use disorder–related inpatient admissions and emergency department visits. Within Medicaid, Keshwani et al^7^ performed the only prospective analysis of buprenorphine PA removals. Using Medicaid State Drug Utilization Data, they evaluated 2 states that removed PAs for all formulations of buprenorphine and found that prescriptions increased in 1 state (Illinois) and were unchanged in the other (California) relative to a control group of states that maintained buprenorphine PAs. More recent work by Landis et al^29^ using Medicaid claims from 2006 to 2013 showed that imposing new buprenorphine PAs was associated with an 11% reduction in the likelihood of retention on medication for 180 days.

Our finding that buprenorphine PA removal was not associated with substantial changes in prescriptions does not match that from Medicare, though it is consistent with the findings of Keshwani et al^7^ that PA removals in Medicaid may have mixed outcomes. Medicaid enrollees with OUD are likely distinct from Medicare enrollees in both their access to buprenorphine treatment and the structural barriers they face. Medicare enrollees are also older, more likely to have a usual source of care, and have increased care continuity compared with Medicaid enrollees.^30,31^ This relative stability in the Medicare population may lead to an increase in buprenorphine prescriptions after PA removal that is not observed in Medicaid enrollees. Furthermore, our subgroup and sensitivity analyses suggest that there may be considerable heterogeneity in the outcomes associated with buprenorphine PA removals across states, with some states (eg, those with low baseline prescribing) possibly experiencing an increase in prescriptions after PA removal that are obscured by the limited changes in other states. Illinois provides an interesting example: similar to Keshwani et al,^7^ our sensitivity analyses suggest that Illinois experienced a considerable increase in buprenorphine prescriptions after its PA removal. Illinois is the only state in our analytic sample that removed PA requirements for all buprenorphine formulations, signifying that the comprehensiveness of PA removal may be an important determinant of the policy effect. Our results also highlight that a complex array of factors along the care cascade may be associated with buprenorphine treatment independent of PA policies, and simply removing PAs may not be enough to overcome entrenched stigma and increase the number of buprenorphine prescribers or patients.

### Limitations

Our study has several limitations. First, state-level data on the number of Medicaid enrollees with OUD were not available during our study period. As such, we are unable to distinguish whether a state’s buprenorphine prescriptions per 1000 Medicaid enrollees is a function of care availability or the proportion of the Medicaid population with OUD. However, we adjusted for state-level overdose death rates as a measure of overall burden of OUD-related effects. Second, we treated buprenorphine PA policies as dichotomous, though the burden of PA policies on prescribers can differ depending on the policy and a given practice’s resources for processing PA requests. Third, we treated the removal of a PA for any formulation of buprenorphine as indicative of PA removal, even though a state may maintain PAs on other formulations of buprenorphine or for higher doses. Fourth, our analyses used state-level data, which may limit our power to detect small but meaningful individual-level associations. Claims data analyses might yield more precise effect estimates and allow for evaluation of PA associations with other important outcomes, such as retention on buprenorphine and health care utilization.^29^ Relatedly, our state-level data did not allow us to identify pregnant people, who are often exempted from PA requirements for buprenorphine, or Medicaid enrollees subject to different prescription benefits (eg, dual-eligible and non–full-benefit enrollees). Follow-up studies using Medicaid claims may reach different conclusions about the association of removing PAs with buprenorphine care. Fifth, our state PA policy classifications came largely from Medicaid FFS plans, which may not be representative of all Medicaid MCO plans within a state.^32^ Sixth, our study may not account for additional barriers that patients may face when trying to maintain buprenorphine prescriptions, including other utilization management policies, such as duration or quantity limits. Seventh, we do not have data on the duration or dosage of buprenorphine prescriptions, which could theoretically confound our estimates if prescription durations increased after PA removal. However, many states maintained limitations on duration and dosage of buprenorphine, even after PA removal, thereby minimizing this limitation.

## Conclusions

In this state-level serial cross-sectional study of Medicaid PA requirements for buprenorphine, removal of PAs was not associated with a substantial change in buprenorphine prescriptions. While there is no clinical or economic justification for limiting access to buprenorphine as a first-line, lifesaving treatment for OUD without a clear alternative that should be trialed first, our results suggest that removing Medicaid buprenorphine PAs alone may not result in substantial increases in buprenorphine prescribing. Given the ongoing burden of opioid overdoses that are driving unprecedented overdose fatality, there is a need for continued multipronged efforts to remove barriers to buprenorphine care and increase availability of this lifesaving treatment.

## Notes

Supplement 1. **eAppendix.** Additional Methods Overview **eFigure 1.** CONSORT Diagram of Analysis of Prior Authorization Removal **eTable 1.** Buprenorphine-Naloxone Medicaid Prior Authorization Status by State as of April 2021 **eFigure 2.** Visual Depiction of Panel Data and Treatment Timing **eFigure 3.** Unadjusted Log Buprenorphine Prescriptions Over Time by State and by Prior Authorization Policy Status, 2015-2019 **eFigure 4.** Visual Depiction of Panel Data and Treatment Timing for Expanded Sample of 40 States Where Medicaid Fee-for-Service (FFS) PA Policies for Buprenorphine Are Not Required to Be the Same **eFigure 5.** Event Study Estimates of Percent Change in Buprenorphine Prescriptions per 1000 Medicaid Enrollees Comparing States That Repealed PAs Versus Those That Maintained PAs in Sensitivity Analysis Restricted to States Where Medicaid FFS PA Policies for Buprenorphine Are Known to Apply to All Medicaid Enrollees and Including Illinois **eFigure 6.** Event Study Estimates of Percent Change in Buprenorphine Prescriptions per 1000 Medicaid Enrollees Comparing States That Repealed PAs vs Those That Maintained PAs in Sensitivity Analysis With Expanded Sample Not Restricted to States Where Medicaid FFS PA Policies for Buprenorphine Are Known to Apply to All Medicaid Enrollees **eFigure 7.** Decomposition of 2 × 2 Difference-in-Differences Estimates and Weights **eFigure 8.** Sensitivity Analyses for the Effect of Prior Authorization Removal on the Mean Difference in Log Buprenorphine Prescriptions per 1000 Medicaid Enrollees Using Different Estimators to Account for Variation in Treatment Timing **eFigure 9.** Generalized Synthetic Control Sensitivity Analyses for the Effect of Prior Authorization Removal on the Mean Difference in Log Buprenorphine Prescriptions per 1000 Medicaid Enrollees **eTable 2.** Summary of Sensitivity Analyses and Point Estimates for the Effect of Prior Authorization Removal on Buprenorphine Prescriptions per 1000 Medicaid Enrollees Using Different Estimators **eTable 3.** Sensitivity Analyses for the Effect of Prior Authorization Removal on Buprenorphine Prescriptions Using Different Exposure Lag Periods **eTable 4.** Sensitivity Analyses for the Effect of Prior Authorization Removal on Buprenorphine Prescriptions per 1000 Medicaid Enrollees Using Different Study Samples and Prior Authorization Categorizations **eTable 5.** National Drug Codes Used for Buprenorphine Prescriptions in the Medicaid State Drug Utilization Database
eReferences

Supplement 2.
Data Sharing Statement
