# Supplementary material for: Removal of Medicaid Prior Authorization Requirements and Buprenorphine Treatment for Opioid Use Disorder
Source: JAMA Health Forum. 2023 Oct 20;4(10):e233549. doi: 10.1001/jamahealthforum.2023.3549 (PMC10589810; doi:10.1001/jamahealthforum.2023.3549)
Supplement: Supplement 1. — eAppendix. Additional Methods Overview eFigure 1. CONSORT Diagram of Analysis of Prior Authorization Removal eTable 1. Buprenorphine-Naloxone Medicaid Prior Authorization Status by State as of April 2021 eFigure 2. Visual Depiction of Panel Data and Treatment Timing eFigure 3. Unadjusted Log Buprenorphine Prescriptions Over Time by State and by Prior Authorization Policy Status, 2015-2019 eFigure 4. Visual Depiction of Panel Data and Treatment Timing for Expanded Sample of 40 States Where Medicaid Fee-for-Service (FFS) PA Policies for Buprenorphine Are Not Required to Be the Same eFigure 5. Event Study Estimates of Percent Change in Buprenorphine Prescriptions per 1000 Medicaid Enrollees Comparing States That Repealed PAs Versus Those That Maintained PAs in Sensitivity Analysis Restricted to States Where Medicaid FFS PA Policies for Buprenorphine Are Known to Apply to All Medicaid Enrollees and Including Illinois eFigure 6. Event Study Estimates of Percent Change in Buprenorphine Prescriptions per 1000 Medicaid Enrollees Comparing States That Repealed PAs vs Those That Maintained PAs in Sensitivity Analysis With Expanded Sample Not Restricted to States Where Medicaid FFS PA Policies for Buprenorphine Are Known to Apply to All Medicaid Enrollees eFigure 7. Decomposition of 2 × 2 Difference-in-Differences Estimates and Weights eFigure 8. Sensitivity Analyses for the Effect of Prior Authorization Removal on the Mean Difference in Log Buprenorphine Prescriptions per 1000 Medicaid Enrollees Using Different Estimators to Account for Variation in Treatment Timing eFigure 9. Generalized Synthetic Control Sensitivity Analyses for the Effect of Prior Authorization Removal on the Mean Difference in Log Buprenorphine Prescriptions per 1000 Medicaid Enrollees eTable 2. Summary of Sensitivity Analyses and Point Estimates for the Effect of Prior Authorization Removal on Buprenorphine Prescriptions per 1000 Medicaid Enrollees Using Different Estimators eTable 3. Sensitivity Analyse [file jamahealthforum-e233549-s001.pdf]

Supplemental Online Content

Christine PJ, Larochelle MR, Lin L, McBride J, Tipirneni R. Removal of Medicaid prior authorization requirements and buprenorphine treatment for opioid use disorder. *JAMA Health Forum*. 2023;4(10):e233549. doi:10.1001/jamahealthforum.2023.3549

eAppendix. Additional Methods Overview

eFigure 1. CONSORT Diagram for Analysis of Prior Authorization Removal

eTable 1. Buprenorphine-Naloxone Medicaid Prior Authorization Status by State as of April 2021

eFigure 2. Visual Depiction of Panel Data and Treatment Timing

eFigure 3. Unadjusted Log Buprenorphine Prescriptions Over Time by State and by Prior Authorization Policy Status, 2015-2019

eFigure 4. Visual Depiction of Panel Data and Treatment Timing for Expanded Sample of 40 States Where Medicaid Fee-for-Service (FFS) PA Policies for Buprenorphine Are Not Required to Be the Same

eFigure 5. Event Study Estimates of Percent Change in Buprenorphine Prescriptions per 1000 Medicaid Enrollees Comparing States That Repealed PAs vs Those That Maintained PAs in Sensitivity Analysis Restricted to States Where Medicaid FFS PA Policies for Buprenorphine Are Known to Apply to All Medicaid Enrollees and Including Illinois

eFigure 6. Event Study Estimates of Percent Change in Buprenorphine Prescriptions per 1000 Medicaid Enrollees Comparing States That Repealed PAs vs Those That Maintained PAs in Sensitivity Analysis With Expanded Sample Not Restricted to States Where Medicaid FFS PA Policies for Buprenorphine Are Known to Apply to All Medicaid Enrollees

eFigure 7. Decomposition of 2 $\times$ 2 Difference-in-Differences Estimates and Weights

eFigure 8. Sensitivity Analyses for the Effect of Prior Authorization Removal on the Mean Difference in Log Buprenorphine Prescriptions per 1000 Medicaid Enrollees Using Different Estimators to Account for Variation in Treatment Timing

eFigure 9. Generalized Synthetic Control Sensitivity Analyses for the Effect of Prior Authorization Removal on the Mean Difference in Log Buprenorphine Prescriptions per 1000 Medicaid Enrolless

eTable 2. Summary of Sensitivity Analyses and Point Estimates for the Effect of Prior Authorization Removal on Buprenorphine Prescriptions per 1000 Medicaid Enrollees Using Different Estimators

eTable 3. Sensitivity Analyses for the Effect of Prior Authorization Removal on Buprenorphine Prescriptions Using Different Exposure Lag Periods

eTable 4. Sensitivity Analyses for the Effect of Prior Authorization Removal on Buprenorphine Prescriptions per 1000 Medicaid Enrollees Using Different Study Samples and Prior Authorization Categorizations

eTable 5. National Drug Codes Used for Buprenorphine Prescriptions in the Medicaid State Drug Utilization Database

eReferences

This supplemental material has been provided by the authors to give readers additional information about their work.

© 2023 Christine PJ et al. *JAMA Health Forum.*

eAppendix. Additional METHODS OVERVIEW

Determination of state Medicaid prior authorization (PA) status
To determine the buprenorphine PA status in each state over time, we undertook an extensive search of formal Medicaid documents including preferred drug lists, state legislative registers, published reports, news reports, and practice bulletin updates. This search was completed in the winter/spring of 2021. Sources for each state are listed in eTable 1 below and include all sources that were used to make the determination. We elected to treat buprenorphine PA status as a binary variable, counting a state as having removed their PA if they made at least one formulation of buprenorphine (including buprenorphine-naloxone) used for opioid use disorder available without a PA. We feel that this is a clinically relevant characterization of PA policies (as opposed to requiring that a state remove all PAs for every buprenorphine formulation[1]), as having access to any formulation of buprenorphine without a PA would theoretically drive providers to utilize that formulation preferentially. In the majority of cases, PAs were removed for a single formulation of buprenorphine at a time, most often buprenorphine sublingual tablets or buprenorphine-naloxone sublingual films. PA classifications were assessed independently by authors McBride and Christine, and any disagreements in classification were discussed and adjudicated with the assistance of author Tipirneni. Even after adjudication, several PA policy changes remained open to interpretation regarding their potential effect on buprenorphine prescribing, and in these cases, we ran additional sensitivity analyses to assess the effect of potential misclassification of the PA policy exposure (see eTable 4).

Buprenorphine prescription data
Our buprenorphine prescription data comes from the Medicaid State Drug Utilization Database. The Urban Institute provided a cleaned version of buprenorphine prescriptions per 1000 Medicaid enrollees, excluding formulations of buprenorphine that are not FDA-approved for opioid use disorder. For additional details on buprenorphine prescription methodology and for downloadable data, see the listed citations.[2,3]

Covariate data sources
We collected data on a variety of state-level characteristics related to OUD burden, sociodemographics, and opioid health policy. We obtained annual opioid overdose rate per 100,000 adults from the CDC WONDER database using Multiple Cause of Death Files.[4] We calculated the annual number of clinicians qualified to prescribe buprenorphine (DEA X-waivered providers) per 100,000 population using data from the Substance Abuse and Mental Health Services Administration and the US Census Bureau. We also obtained the number of individuals receiving methadone through an opioid treatment program (i.e. a methadone clinic) per 100,000 adults from the National Survey of Substance Abuse Treatment Services.[5] Percent of individuals aged 12 and older living below the federal poverty limit was available annually from the US Census Bureau's Small Area Income and Poverty Estimates Program.[6] We used the Kaiser Family Foundation briefs for information on state Medicaid expansion status and the proportion of individuals on Medicaid covered under comprehensive managed care plans.[7,8] Finally, we used data from Lee and colleagues to classify states according to whether or not they mandated use of a prescription drug monitoring program (PDMP) for prescribing opioids.[9]

Sample restriction to states where Medicaid Fee for Service (FFS) PA policies for buprenorphine apply to all Medicaid enrollees
The majority of our PA policy data came from states' Medicaid FFS plans. Complete data on Medicaid Managed Care Organization (MCO) PA policies is not available during our study period. In states with substantial numbers of MCO enrollees, if PA policies for buprenorphine differ in MCO vs FFS plans, this may bias the effect estimates in our main models. To account for this possibility, we used data from CMS's annual Medicaid Drug Utilization Review reports to restrict our main analytic sample to states where Medicaid FFS PA policies for buprenorphine are known to apply to all Medicaid enrollees.[10] This sample includes states that meet at least one of the following criteria throughout the study period: 1) the state has no Medicaid MCOs (i.e. is all FFS); 2) the state MCO pharmacy benefits are carved out to FFS Medicaid; 3) the state MCO utilization management policies must either follow or be no more restrictive than FFS policies (as indicated in the Drug Utilization Review reports). After applying these restriction criteria, 24 states were available for analysis. Because Illinois included limited pre-period data and was the only state contributing to effect estimates after quarter 9 in the post-period, we also excluded Illinois in our main analyses. To assess the sensitivity of our effect estimates to different sample restrictions, we reran the same event study and two-way fixed effects models as above with two expanded samples: a) including Illinois (n=24 states, see

© 2023 Christine PJ et al. *JAMA Health Forum.*

eFigure 5), and b) including Illinois and states that allowed PA policies for buprenorphine to differ between Medicaid FFS and MCO plans (n=40 states, see eFigure 4 and eFigure 6).

Main statistical analyses

Two-way fixed effects models: For our main two-way fixed effects (TWFE) difference-in-difference analyses, we estimated a log-linear regression model at the state-quarter level. For state *i*, in quarter *t*, we fitted:

$$(1)\ Log\ Bup\ Rx_{it} = \beta_1 PA_{it} + \beta_2 X_{it} + \beta_3 State_i + \beta_4 Year_t + \varepsilon_{it}$$

where $Log\ Bup\ Rx_{it}$ is the log number of buprenorphine prescriptions per 1000 Medicaid enrollees; PA_{it} is 1 if a state removed their Medicaid prior authorization requirement for at least one formulation of buprenorphine, 0 otherwise; X_{it} is a vector of time-varying covariates: opioid overdose rate per 100,000 adults, log number of X-waivered clinicians qualified to prescribe buprenorphine per 100,000 population, number of individuals receiving methadone through an opioid treatment program per 100,000 adults (including quadratic term); percent of individuals aged 12 and older living below the federal poverty limit, Medicaid expansion status, percent of Medicaid enrollees covered under comprehensive managed care plans, and prescription drug monitoring program mandate (1 if yes, 0 if no); $State_i$ and $Year_t$ represent state and year fixed effects; and ε_{it} is an error term. Because models with quarter fixed effects showed very similar results to those with year fixed effects, we utilized year fixed effects in the final version of our model to improve statistical power. Standard errors were clustered at the state level. The treatment effect for removing a buprenorphine prior authorization is given by the parameter β_1. To ease interpretation, effect estimates were exponentiated and can therefore be interpreted as the percent change in buprenorphine prescriptions per 1000 Medicaid enrollees after removing prior authorization, controlling for all other covariates in the model.

Difference-in-difference-in-differences ("Triple Difference") models: For models evaluating whether the effect of prior authorization removal differed according to baseline state characteristics, we estimated a log-linear regression model at the state-quarter level. For state *i*, in quarter *t*, we fitted:

$$(2)\ Log\ Bup\ Rx_{it} = \beta_1 PA_{it} + \beta_2 X_{it} + \beta_3 State_i + \beta_4 Year_t + \beta_5 Base_i + \beta_6 PA_{it} * Base_i + \varepsilon_{it}$$

where all variables are the same as those listed above in equation 1, aside from *Base* which corresponds to baseline (i.e. quarter 1 of 2015) state-level variables hypothesized *a priori* to potentially modify the effect of prior authorization removal of buprenorphine prescriptions, including above vs below-median baseline buprenorphine prescriptions per 1000 Medicaid enrollees, Medicaid expansion status, and above vs below-median percent of Medicaid enrollees covered under comprehensive managed care plans. We again utilized year fixed effects in the final version of our models to improve statistical power. Standard errors were clustered at the state level. The parameter of interest was β_6 which represents the difference in log buprenorphine prescriptions per 1000 Medicaid enrollees after removing a buprenorphine prior authorization in states with various levels of the baseline characteristics. Estimates were again exponentiated for ease of interpretation and can therefore be interpreted as the percent change in buprenorphine prescriptions per 1000 Medicaid enrollees, controlling for all other covariates in the model.

Bacon decomposition

The canonical two-way fixed effects (TWFE) model relies on several assumptions to identify the average treatment effect on the treated (ATT), including that the average outcome in both the treated and comparison groups would have followed parallel trends in the post-period even in the absence of treatment. Recent work has demonstrated that issues can arise with TWFE models when there are multiple time periods with variation in treatment timing.[11,12] In these cases, the coefficients from a TWFE regression model may not be a weighted average of unit-level treatment effects as anticipated. Rather, in the setting of heterogeneous treatment effects, TWFE models can include desired comparisons between treated and not-yet-treated units, but also undesired comparisons between treated and previously treated units that can lead to biased estimates. To diagnose whether such issues are present in our study, we use the decomposition method developed by Goodman-Bacon.[12] In short, this decomposition method allows us to visualize the various treatment group comparisons that give rise to our effect estimates, as well as the effect size and weight assigned to each treatment group comparison (see eFigure 7).

Sensitivity analyses to account for differential treatment timing

As highlighted above, TWFE models may produce biased treatment effect estimates in the setting of multiple time periods with variation in treatment timing. While the Bacon decomposition described above can help diagnose how big of an issue this is in our study, alternative estimators have recently been proposed that seek to minimize/eliminate the biases caused by variation in treatment timing.[11] We employ two of these estimators to assess whether our findings are robust to these different approaches.

First, we use the estimator proposed by Callaway and Sant'Anna which calculates group-time average treatment effects using not-yet-treated states as the control group.[13] This estimator excludes undesired state comparisons, which in our case would occur if a state that repealed a buprenorphine PA in a later year was compared to a state that repealed a buprenorphine PA in an earlier year. We employ this estimator in an event study framework controlling for opioid overdose deaths per 100,000 residents and Medicaid expansion status (additional covariate inclusion resulted in more extreme inverse probability of treatment weights) and calculate an overall ATT which averages treatment effects across all lengths of exposure to treatment (see eFigure 8 and eTable 2).

Second, we use the estimator proposed by Sun and Abraham which similarly excludes undesired state comparisons but uses last-to-be-treated states as the control group rather than not-yet-treated states as in the Callaway and Sant-Anna estimator.[14] We again employ this estimator in an event study framework controlling for all covariates included in our main models, and calculate an overall ATT (see eFigure 8 and eTable 2).

Generalized synthetic control sensitivity analyses

As an alternative to the traditional difference-in-differences framework, we also employed a generalized synthetic control (GSC) method to estimate the effect of removing buprenorphine PA requirements. This method does not impose the parallel trends assumption required in difference-in-difference models and may better account for unobserved heterogeneity between treatment and control states.[15] Synthetic control approaches reweight and combine information from control units into a "synthetic control" that matches the treated units in the pre-intervention period. The resulting synthetic control unit is then used to predict counterfactual outcomes in the post-treatment period which are then compared to the treated units to generate an effect estimate.[16] The GSC method is an extension of the traditional synthetic control method that allows for multiple treated units and variation in treatment timing, as is the case with buprenorphine PA repeals. We employ the GSC model proposed by Xu which uses an interactive fixed effects model to predict post-PA repeal counterfactual buprenorphine prescriptions.[17] We use a parametric bootstrap procedure clustered at the state level with 1000 iterations to generate confidence intervals. Models with and without time-varying covariates produced very similar results, so we present simplified models without time-varying covariates (see eFigure 9). Rhode Island and Illinois were excluded from analyses due to limited pre-period data.

Relevant R packages used

Decomposition of 2x2 difference in differences (Bacon decomposition): *bacon v0.1.1*
Two-way fixed effects models; Sun and Abraham estimator: *fixest v0.10.1*
Callaway and Sant'Anna estimator: *did v2.1.0*
Plots of different event study estimators: *did2s v0.7.0*
Generalized synthetic control: *gsynth v1.2.1*

© 2023 Christine PJ et al. *JAMA Health Forum.*

eFigure 1. CONSORT Diagram for Analysis of Prior Authorization Removal

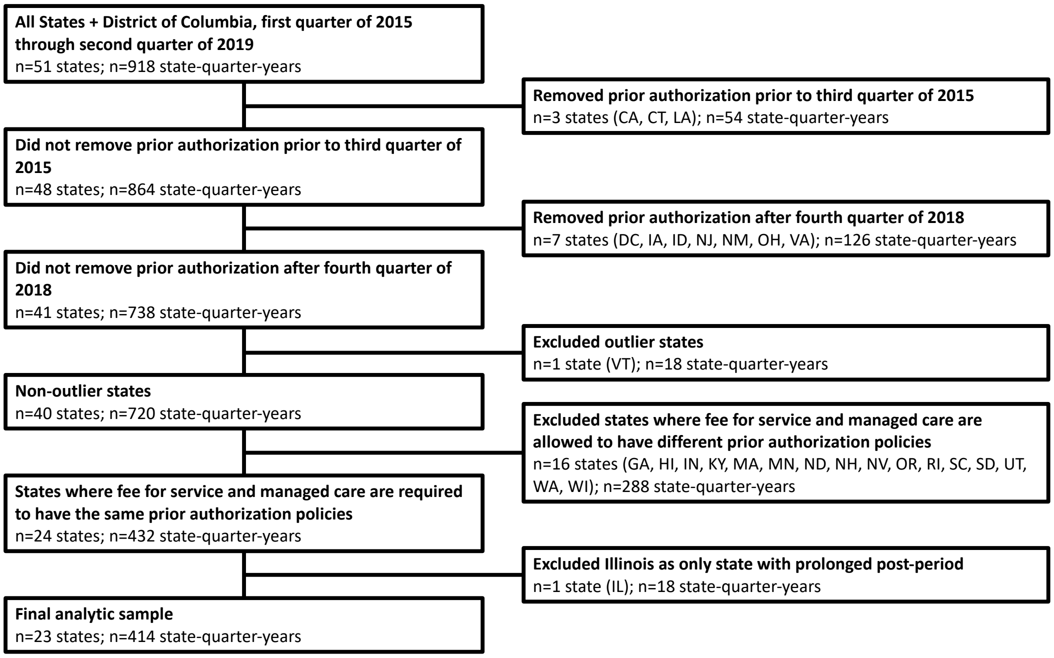

© 2023 Christine PJ et al. *JAMA Health Forum.*

eTable 1. Buprenorphine Medicaid Prior Authorization Status by State as of April 2021[1]

State	PA Removal Status Used in Main Analyses (Q1 2015-Q2 2019)	PA Removal Status as of April 2021	Removal Date (Effective Date – if different than removal date)[2]	Description	Sources
Alabama	No	No		Requires PA for all forms of buprenorphine-naloxone.	[3]
Alaska[4]	No	No		PA not required for first 28-days of treatment but required thereafter.	[5,6]
Arizona	Yes	Yes	January 2018	PA not required for buprenorphine-naloxone sublingual films. PA still required for long-acting buprenorphine-naloxone.	[7,8,9,10]
Arkansas[11]	No	Yes	April 2019 (January 2020)	New law passed in April 2019 that prohibits PAs for medications for opioid use disorder. Law takes effect January 2020.	[12,13]
California	Excluded	Yes	June 2015	PA not required by Medi-Cal for the treatment of opioid use disorder	[14,15,16]
Colorado	No	Yes	January 2020	PA removed by Colorado Medicaid, and managed care organizations prohibit PA for any FDA-approved medications for SUD	[17,18]

[1] PA policies were reviewed in April 2021. Additional changes may have occurred since that time. Please see eAppendix 1 for further discussion of differences in PA classification methodology.
[2] Some states repealed PA on a given date, but policy was not effective until another date. Effective date was used as the policy change date in all analyses.
[3] https://medicaid.alabama.gov/documents/4.0_Programs/4.3_Pharmacy-DME/4.3.7_Preferred_Drug_List/4.3.7_PDL_Thera_4-1-21.pdf
[4] Alaska repealed requirement for PA for first 28-days of prescription but required for all fills thereafter. We treat Alaska as a control state in the main analyses, with a sensitivity analysis treating Alaska as removing their PA in April 2017. See table of sensitivity analyses below in supplement.
[5] http://dhss.alaska.gov/dhcs/Documents/pharmacy/Criteria/Oral_buprenorphine-PA-20180921.pdf
[6] http://manuals.medicaidalaska.com/docs/dnld/Update_Pharmacy_Sept_2018.pdf
[7] https://www.azahcccs.gov/Resources/Downloads/PharmacyUpdates/AHCCCSDrugList_20210101.pdf
[8] https://www.azcentral.com/story/money/business/health/2018/04/21/battle-beat-opioid-addiction-arizonas-medicaid-program-favors-one-drug/534231002/
[9] https://www.azahcccs.gov/PlansProviders/Downloads/PharmacyUpdates/AHCCCSPreferredDrugs_01_01_2018. pdf
[10] https://law.justia.com/codes/arizona/2018/title-20/section-20-3402/
[11] Arkansas repealed PA through law passed in April 2019, but policy is not effective until January 2020.
[12] https://www.arkleg.state.ar.us/Acts/Document?type=pdf&act=964&ddBienniumSession=2019%2F2019R
[13] https://healthcarejournalar.com/sites/default/files/journal/fb/HJAR-2020-nov-dec/43/
[14] https://medi-calrx.dhcs.ca.gov/cms/medicalrx/static-assets/documents/provider/forms-and-information/cdl/Medi-Cal_Rx_Contract_Drugs_List_FINAL.pdf
[15] https://files.medi-cal.ca.gov/pubsdoco/dur/Articles/dured_25096.pdf
[16] https://khn.org/news/use-of-buprenorphine-to-treat-opioid-addiction-proliferates-in-california/
[17] https://leg.colorado.gov/bills/hb19-1269
[18] https://www.colorado.gov/pacific/sites/default/files/01-01-21%20PDL..v2.pdf

© 2023 Christine PJ et al. *JAMA Health Forum.*

Connecticut	Excluded	Yes	April 2010	Buprenorphine-naloxone sublingual film has been on preferred drug list since without a PA since April 2010	19,20
Delaware[21]	Yes	Yes	January 2017 (July 2017)	Buprenorphine-naloxone added to PDL without prior authorization, along with other preferred agents for OUD. PA was repealed in January 2017 but not actively implemented until July 2017.	22,23,24
District of Columbia	Excluded	Yes	April 2019	Removed PA for all FDA-approved medications for OUD, except for buprenorphine-naloxone doses higher than 24mg/day.	25,26,27
Florida[28]	No	No		Require PA for all buprenorphine-naloxone formulations. There is an exception for a 7-day supply without a PA if a patient has not been on MOUD in the past 12 months, with the option of two 7-day refills within a two-month span.	29,30,31,32,33
Georgia	Excluded	Yes	November 2017	PA removed by fee-for-service Medicaid for buprenorphine-naloxone tablets (preferred form). PA still required for other non-preferred forms.	34,35
Hawaii[36]	Excluded	Yes	June 2017	Hawaii Medicaid largely run by managed care organizations. PA removed in June 2017 from the largest Medicaid managed care organization.	37,38,39

[19] https://www.ctdssmap.com/CTPortal/Portals/0/StaticContent/Publications/CT_PDL_medicaid.pdf
[20] https://www.ctdssmap.com/CTPortal/Pharmacy%20Information/tabId/65/Default.aspx
[21] Policy changed in January 2017 but per the cited bulletin, though did not take effect until July 2017.
[22] https://medicaidpublications.dhss.delaware.gov/docs/DesktopModules/Bring2mind/DMX/API/Entries/Download?Command=Core_Download&EntryId= 940&language=en-US&PortalId=0&TabId=94
[23] https://www.dhss.delaware.gov/dhss/dmma/files/buprenorphine_naloxone_prescription_review.pdf
[24] https://delcode.delaware.gov/title31/c005/index.html
[25] https://dc.fhsc.com/downloads/providers/DCRx_PDL_listing.pdf
[26] https://dhcf.dc.gov/sites/default/files/dc/sites/dhcf/publication/attachments/Policy%20%2319-001%20Removal%20of%20Prior%20Auth.%20Req.%20for%20Medication-Assis_0.pdf
[27] https://dhcf.dc.gov/sites/default/files/dc/sites/dhcf/publication/attachments/Final%20Policy%20Access%20to%20Buprenorphine.pdf
[28] Because Florida does provide an exception for a 7-day prescription without a PA, we perform a sensitivity analysis reclassifying Florida as repealing its PA in February 2018.
[29] https://ahca.myflorida.com/medicaid/prescribed_drug/banners.shtml
[30] https://ahca.myflorida.com/medicaid/Prescribed_Drug/pharm_thera/pdf/PDL.pdf
[31] https://ahca.myflorida.com/medicaid/prescribed_drug/pharm_thera/paforms/Buprenorphine_Agents_Form.pdf
[32] https://ahca.myflorida.com/medicaid/Prescribed_Drug/pdf/FL_Medicaid_Brand_Preferred_List.pdf
[33] https://health.wusf.usf.edu/health-news-florida/2018-02-21/medicaid-program-limiting-opioid-prescriptions
[34] https://dch.georgia.gov/providers/provider-types/pharmacy/preferred-drug-lists
[35] https://dch.georgia.gov/providers/provider-types/pharmacy/preferred-drug-lists/2017-preferred-drug-list
[36] Data for Hawaii is difficult to locate, but the state's largest Medicaid managed care organization did repeal their PA in June 2017, which we use as our date of repeal. SAMHSA reports from 2014 and 2018 also indicate that the PA was removed between those years.
[37] https://medquest.hawaii.gov/en/plans-providers/pharmacy/drug-coverage/formulary-drug-search.html?searchNDC=00054018913
[38] https://www.capitol.hawaii.gov/session2018/bills/SB2269_.HTM
[39] https://medquest.hawaii.gov/en/search.html?q=prior+authorization+SUBOXONE

© 2023 Christine PJ et al. *JAMA Health Forum.*

Idaho	Excluded	Yes	May 2019	Removed PA for buprenorphine-naloxone tablets in May 2019 according to P&T committee notes and archived PDLs.	[40,41,42,43]
Illinois	Excluded	Yes	July 2015	Medicaid fee for service and managed care programs cover FDA-approved medications for OUD without a PA.	[44]
Indiana	Excluded	Yes	December 2017	Repealed PA requirement for buprenorphine-naloxone tablets. Still requires PA for films.	[45,46]
Iowa	Excluded	Yes	May 2019	Law passed requiring that Medicaid must cover one form of medication for opioid use disorder without a prior authorization.	[47,48]
Kansas	No	No		Requires PA for buprenorphine-naloxone with documentation of clinical criteria for medication and behavioral treatment participation.	[49,50,51]
Kentucky	Excluded	Yes	January 2020	Repealed PA requirement for preferred buprenorphine-naloxone products.	[52,53,54]
Louisiana[55]	Excluded	Yes	July 2011	Repealed PA for brand-name buprenorphine-naloxone films (Suboxone) as evidenced though archived PDLs. Still required PA for generic buprenorphine-naloxone films until 2021.	[56,57,58]

[40] https://publicdocuments.dhw.idaho.gov/WebLink/DocView.aspx?id=3045&dbid=0&repo=PUBLIC-DOCUMENTS
[41] https://publicdocuments.dhw.idaho.gov/WebLink/DocView.aspx?id=15075&dbid=0&repo=PUBLIC-DOCUMENTS&cr=1
[42] https://www.rsat-tta.com/Files/MAT-Medicaid-Review_updated-12-2019
[43] https://publicdocuments.dhw.idaho.gov/WebLink/DocView.aspx?id=15075&dbid=0&repo=PUBLIC-DOCUMENTS&searchid=d2a34ec2-563c-4fdc-b4ff-cab5421bb5ab
[44] https://www.ilga.gov/legislation/fulltext.asp?DocName=10200HB2589sam001&GA=102&LegID=131409&SessionId=110&SpecSess=0&DocTypeId=HB&DocNum=2589&GAID=16&Session=
[45] https://prdgov-rxadmin.optum.com/rxadmin/INM/20210401_INM_PDL_1.pdf
[46] https://www.wfyi.org/news/articles/indiana-just-made-getting-addiction-treatment-a-little-bit-easier
[47] http://www.iowamedicaidpdl.com/sites/default/files/ghs-files/2020-12-02/ia-web-pdl_jan2021_final.pdf
[48] https://legiscan.com/IA/bill/HF623/2019
[49] https://www.kdheks.gov/hcf/pharmacy/download/PDLList.pdf
[50] https://www.kdheks.gov/hcf/pharmacy/PA_Criteria/Clinical_PA_Table_of_Contents.pdf
[51] https://www.kdheks.gov/hcf/pharmacy/PA_Criteria/Opioid_PA_Criteria.pdf
[52] https://kyportal.magellanmedicaid.com/public/client/static/kentucky/documents/PreferredDrugGuide_full.pdf
[53] https://mediprovider.anthem.com/dam/publicdocuments/KYKY_CAID_BuprenorphinePAForm.pdf
[54] https://apps.legislature.ky.gov/record/19RS/sb54.html#actions
[55] Policy HR 257 proposed that the Department of Health eliminate PAs in Medicaid for all formulations of buprenorphine-naloxone and naltrexone in 2019. Through reviewing prior PDLs, brand-name buprenorphine-naloxone films (Suboxone) have been on the PDL without a PA since July 2011.
[56] https://ldh.la.gov/assets/HealthyLa/Pharmacy/PDL.pdf
[57] https://www.lamedicaid.com/provweb1/forms/pharmacy_archived_forms.htm
[58] https://ldh.la.gov/assets/docs/LegisReports/HR257RS201912020.pdf#:~:text=HR%20257%20of%2

© 2023 Christine PJ et al. *JAMA Health Forum.*

Maine	No	Yes	June 2020	Policy changed in June 2020 requiring at least one medication for OUD from each therapeutic class to be covered without a PA. For pregnant persons, all medications for OUD are covered without a PA.	[59,60]
Maryland	Yes	Yes	May 2017	PA not required for brand-name buprenorphine-naloxone films (Suboxone) since May 2017.	[61,62]
Massachusetts	Excluded	Yes	July 2019	PA not required for preferred brand-name buprenorphine-naloxone films (Suboxone), though still requiring for non-preferred formulations and for all doses >32mg/day or >24mg/day for longer than 90 days.	[63,64,65]
Michigan[66]	No	Yes	December 2019	PA removed for sublingual tabs and films for preferred products. Generic buprenorphine-naloxone films still require PA.	[67,68,69]
Minnesota	Excluded	Yes	August 2018	PA repealed for preferred buprenorphine-naloxone formulation.	[70,71,72,73]
Mississippi[74]	No	Yes	July 2019	Mississippi employs an automated PA service that occurs when a prescription for buprenorphine-naloxone is written. This automated service checks to make sure that a diagnosis of OUD is documented, and if it is,	[75,76,77,78]

[59] http://mainecarepdl.org/sites/default/files/ghs-files/pdl/2021-04-20/pdlmainecriteria-042021.pdf
[60] http://legislature.maine.gov/legis/bills/bills_129th/billtexts/HP137801.asp
[61] https://mmcp.health.maryland.gov/pap/docs/PDL%201.1.21_3.1.2021.pdf
[62] https://legiscan.com/MD/text/HB887/id/1627694
[63] https://mhdl.pharmacy.services.conduent.com/MHDL/pubdruglist.do?category=MassHealth+Drug+List+A+-+Z
[64] https://mhdl.pharmacy.services.conduent.com/MHDL/pubdownloadpa.do?id=5524
[65] https://www.mass.gov/doc/issue-1-january-2020-0/download
[66] Michigan initially made preferred medications for OUD available without a PA for an intial-14-day supply starting in May 2019. A PA was initially still required after the first 14-day fill, but this PA requirement was fully removed in December 2019. Using either date, Michigan is treated as a control state in our analysis as it had a PA throughout the period of interest.
[67] https://bhbusiness.com/2019/12/05/another-state-removes-prior-authorization-for-medication-assisted-treatment/
[68] https://michigan.magellanrx.com/provider/external/medicaid/mi/doc/en-us/MIRx_PDL.pdf
[69] https://michigan.magellanrx.com/provider/external/medicaid/mi/doc/en-us/MIRx_MAT_FAQ.pdf
[70] https://www.gao.gov/assets/gao-20-233.pdf
[71] https://mn.gov/dhs/assets/preferred-drug-list-2021-01-01_tcm1053-456912.pdf
[72] https://mn.gov/dhs/assets/dhs-1115-mat-policy-statement_tcm1053-451090.pdf
[73] https://mn.gov/dhs/partners-and-providers/policies-procedures/minnesota-health-care-programs/provider/types/rx/pa-criteria/buprenorphine-naloxone.jsp
[74] Mississippi changed to an automated PA service in July 2019 that automatically checks whether a patient has a diagnosis of OUD documented, and if they do, the prescription (for preferred forms of buprenorphine-naloxone) is approved. This process reportedly occurs rapidly and requires no specific form to be completed by providers. As such, it is open for interpretation whether or not Mississippi should be considered to have a PA in the traditional sense. For our analysis, Mississippi is treated as a requiring a PA, as this was the case throughout our study period.
[75] https://medicaid.ms.gov/wp-content/uploads/2014/04/Enhanced-Prior-Authorization-Program-for-Medications.pdf
[76] https://medicaid.ms.gov/providers/pharmacy/preferred-drug-list/preferred-drug-list-archive/
[77] https://medicaid.ms.gov/wp-content/uploads/2019/11/DURAgenda120519.pdf
[78] http://www.medicaid.ms.gov/wp-content/uploads/2014/04/BuprenorphineNaloxoneBuprenorphineSummaryProviders.pdf

© 2023 Christine PJ et al. *JAMA Health Forum.*

				approves the prescription. If no diagnosis is documented, the prescription is rejected.	
Missouri	No	Yes	August 2019	PA repealed for preferred formulation of brand-name buprenorphine-naloxone film (Suboxone). PA still required for non-preferred formulations.	[79,80]
Montana[81]	No	No		PA required for buprenorphine-naloxone products.	[82,83,84,85]
Nebraska	Yes	Yes	November 2017	PA repealed for preferred formulations of buprenorphine-naloxone.	[86,87,88]
Nevada	Excluded	Yes	January 2020	PA repealed for preferred formulations of buprenorphine-naloxone and buprenorphine monotherapy.	[89,90,91,92]
New Hampshire	Excluded	No		PA still required for buprenorphine-naloxone products.	[93,94,95]
New Jersey[96]	Excluded	Yes	April 2019	PA repealed for all FDA-approved medications for OUD	[97,98,99]
New Mexico[100]	Excluded	Yes	April 2019	PA repealed for preferred formulations of buprenorphine-naloxone.	[101,102,103,104]

[79] https://pharmacy.services.conduent.com/mohealthnet/19_3_MOHealthNet%20PDL%20and%20Diabetic%20Supply%20Preferred%20Drug%20List%20Forms/21_03%20PDL%20DOCS/Posting%20PDL%20Static%20Document_3.1.2021.pdf
[80] https://revisor.mo.gov/main/OneSection.aspx?section=191.1165
[81] Montana has passed a law outlawing PAs for OUD in commercial insurance plans, but this does not appear to be the case for Medicaid plans.
[82] https://medicaidprovider.mt.gov/docs/forms/Buprenorphine-containingproductsPAFormtransmucosal04052021.pdf
[83] https://leg.mt.gov/bills/2019/BillHtml/HB0555.htm
[84] https://medicaidprovider.mt.gov/Portals/68/docs/forms/BuprenorphinecontainingproductsPAForm11062017.pdf
[85] https://medicaidprovider.mt.gov/Portals/68/docs/pharmacy/2021pharm/PDLJanuary152021.pdf
[86] https://nebraska.fhsc.com/downloads/PDL/NE_PDL-20210401.pdf
[87] https://nebraska.fhsc.com/Downloads/NEfaxform-Suboxone.pdf
[88] https://dhhs.ne.gov/Documents/DUR%20Newsletter%20Jan%202018.pdf
[89] https://dhcfp.nv.gov/uploadedFiles/dhcfpnvgov/content/Pgms/CPT/InformationalBulletinOnMedicationsAndServicesForSubstanceUseDisorders.pdf
[90] https://www.medicaid.nv.gov/Downloads/provider/FA-73.pdf
[91] https://www.medicaid.nv.gov/Downloads/provider/NV_PDL_20200102.pdf
[92] https://www.medicaid.nv.gov/providers/rx/PDL.aspx
[93] https://www.dhhs.nh.gov/ombp/pharmacy/documents/preferred.pdf
[94] https://www.dhhs.nh.gov/ombp/pharmacy/documents/buprenorphinecriteria.pdf
[95] https://nhcontent.magellanmedicaid.com/Downloads/provider/NHRx_PA_fax_buprenorphine.pdf
[96] New Jersey passed official legislation removing all PAs for buprenorphine-naloxone as of October 15, 2019. However, the New Jersey Department of Health and Human Services noted that the PA requirement policy was removed as of April 1, 2019.
[97] https://www.nj.gov/humanservices/news/pressreleases/2019/approved/20190401.html
[98] https://whyy.org/articles/access-to-treatment-for-opioid-addiction-eased-for-those-on-medicaid-in-nj/
[99] https://fm.formularynavigator.com/FBO/4/New_Jersey_PDL_English.pdf
[100] We use archived PDLs from several MCOs indicating that the largest MCO repealed their PA for buprenorphine-naloxone in April 2019.
[101] http://docs.phs.org/idc/groups/public/documents/communication/pel_00199170.pdf
[102] https://www.westernskycommunitycare.com/content/dam/centene/newmexico/Medicaid/PDFs/NM%20CNCNM001_20190227_Final%20PDF%20508C. PDF
[103] https://www.hsd.state.nm.us/wp-content/uploads/2020/12/BH-POLICY-AND-BILLING-MASTER_.pdf
[104] https://www.bcbsnm.com/community-centennial/pdf/cc-drug-list-nm.pdf

© 2023 Christine PJ et al. *JAMA Health Forum.*

New York[105]	No	No		PA still required for buprenorphine-naloxone products as of April 2021.	106,107,108, 109
North Carolina	Yes	Yes	January 2018	PA repealed for preferred formulations of brand name buprenorphine-naloxone films (Suboxone). PA still required for buprenorphine monotherapy formulations.	110,111
North Dakota	Excluded	Yes	January 2020	PA repealed for buprenorphine-naloxone tablets. PA still required for buprenorphine-naloxone films.	112
Ohio	Excluded	Yes	January 2019	PA repealed for preferred formulations of buprenorphine-naloxone products. PA still required for non-preferred formulations.	113,114
Oklahoma[115]	No	Yes	July 2019	PA repealed for generic buprenorphine-naloxone tablets. PA still required for films and non-generic formulations.	116,117,118, 119
Oregon[120]	Excluded	Yes	April 2017	PA removed for generic buprenorphine-naloxone tablets first, then removed for films at a later date.	121,122,123
Pennsylvania	Yes	Yes	March 2018	PA removed for preferred generic buprenorphine-naloxone tablet/film. PA still required for non-preferred formulations.	124,125,126

[105] New York passed Act A.9271 in 2016 which mandated that Medicaid managed care plans follow the state's fee for service PDL. It also stipulated that PAs could not be required for various conditions, including "mental illness" if it was recommended by the pharmacy committee. The act did not specify OUD or addiction as qualifying conditions. According to various articles in the press, the PA for buprenorphine products was not removed in Medicaid until March 2022.
[106] https://filtermag.org/hochul-medication-opioid-use-disorder-medicaid-bill/?mc_cid=a90969e5e3&mc_eid=8948b46559
[107] https://www.nysenate.gov/legislation/bills/2019/s4808
[108] https://www.psychcongress.com/article/new-york-advocates-win-one-lose-another-prior-authorization
[109] https://assembly.state.ny.us/leg/?default_fld=&leg_video=&bn=A09271&term=2015&Summary=Y&Text=Y
[110] https://files.nc.gov/ncdma/documents/files/1A-41_4.pdf
[111] https://www.nctracks.nc.gov/content/public/providers/pharmacy/pa-drugs-criteria-new-format.html
[112] http://hidesigns.com/assets/files/ndmedicaid/2021/PDL_2021.3.pdf
[113] https://pharmacy.medicaid.ohio.gov/pa-criteria-update-history
[114] https://medicaid.ohio.gov/Portals/0/Resources/Publications/Guidance/Increasing-access-to-Medication-Assisted-Treatment.pdf
[115] Oklahoma repealed their PA in July 2019. Prior to that time, PA was required for everyone except pregnant people.
[116] https://oklahoma.gov/content/dam/ok/en/okhca/documents/a0302/23629.pdf
[117] https://www.dccca.org/wp-content/uploads/OHCA-Changes-and-Review-for-Access-to-Treatment-Through-Medicaid.pdf
[118] https://oklahoma.gov/content/dam/ok/en/okhca/documents/a0302/23638.pdf
[119] https://oklahoma.gov/ohca/providers/types/pharmacy/prior-authorization/2022/central-nervous-system-behavioral-health.html
[120] Oregon passed bill OR HB2257 on August 23, 2019 which expressly prohibits prior authorizations for medications for substance use disorders for the first 30-days of treatment. However, we find that the PA for buprenorphine-naloxone generic tablets was removed in April 2017 in fee for service Medicaid, though quantity limits still applied. We use the April 2017 date in our sensitivity analysis that includes Oregon.
[121] https://www.oregon.gov/OHA/HSD/OHP/Pages/PDL.aspx?wp6271=l:100
[122] https://www.oregon.gov/oha/HSD/OHP/Tools/Oregon%20Medicaid%20Preferred%20Drug%20List,%20April%201,%202021.pdf
[123] https://www.billtrack50.com/BillDetail/1011831#:~:text=bill%20summary,-Chamber&text=H-
[124] https://papdl.com/sites/default/files/ghs-files/Penn%20Statewide%20PDL%2001.05.21.pdf
[125] https://whyy.org/segments/pennsylvania-cuts-red-tape-medication-assisted-treatment-opioid-addiction/
[126] https://www.media.pa.gov/Pages/Insurance-Details.aspx?newsid=344

© 2023 Christine PJ et al. *JAMA Health Forum.*

Rhode Island	Excluded	Yes	September 2015	PA removed for brand name buprenorphine-naloxone films (Suboxone). PA still required for non-preferred formulations.	[127]
South Carolina	Excluded	Yes	July 2018	PA repealed for preferred buprenorphine-naloxone formulations. PA still required for buprenorphine monotherapy formulations. Quantity limits apply.	[128,129,130]
South Dakota[131]	Excluded	Yes	October 2019	PA repealed for brand name buprenorphine-naloxone films (Suboxone). PA still required for non-preferred formulations.	[132]
Tennessee	No	No		PA still required for all formulations of medications for OUD.	[133,134,135]
Texas[136]	No	No		PA still required for buprenorphine-naloxone products.	[137,138]
Utah	Excluded	Yes	April 2018	PA removed for preferred buprenorphine-naloxone formulations for first 180 days of treatment.	[139]
Vermont[140]	Excluded	Yes	October 2018	PA removed for preferred buprenorphine-naloxone formulations though still required for doses over 16mg/day.	[141,142]
Virginia	Excluded	Yes	January 2019	PA removed for preferred buprenorphine-naloxone formulations.	[143,144]

[127] https://eohhs.ri.gov/sites/g/files/xkgbur226/files/2021-03/pdl_list.pdf
[128] https://southcarolina.fhsc.com/Downloads/provider/SCpdl_listing_20210215.pdf
[129] http://southcarolina.fhsc.com/Downloads/provider/SCRx_Reference_Guide_MAT.pdf
[130] http://southcarolina.fhsc.com/Downloads/provider/PTMinutes_20180606.pdf
[131] Note that South Dakota does not make their PDLs public, and pharmacy benefits are managed through Optum Rx.
[132] https://dss.sd.gov/docs/medicaid/advisorycommittee/10.23.19/Medicaid_Opioid_Edits.pdf
[133] https://www.optumrx.com/content/dam/openenrollment/pdfs/Tenncare/home-page/preferred-drug-list/Preferred%20Drug%20List%20(PDL).pdf
[134] https://www.optumrx.com/content/dam/openenrollment/pdfs/Tenncare/presciber/clinical-criteria/Criteria%20PDL.pdf#nameddest=buprenorphine_naloxone
[135] https://www.optumrx.com/content/dam/openenrollment/pdfs/Tenncare/presciber/prior-authorization-forms/Buprenorphine%20Products%20PA%20Form.pdf
[136] Texas passed a bill on September 1, 2019 that states that PAs should be removed except as deemed necessary to prevent fraud and abuse. It appears the bill left ultimate authority up to the board of pharmacy, and per our review of numerous pharmacy documents for the various Texas Medicaid plans (fee for service and managed care plans), PAs are still required for buprenorphine products in all plans except for one. We therefore classify Texas as still requiring a PA, though classification in our study sample would not alter the results given the timing of the law’s passage.
[137] https://www.txvendordrug.com/formulary/prior-authorization/ffs-clinical-pa
[138] https://www.txvendordrug.com/sites/default/files/docs/cpa-assistance-chart.pdf
[139] https://medicaid.utah.gov/pharmacy/preferred-drug-list
[140] Vermont’s PA was repealed for preferred formulations in October 2018, though quantity and dose limits apply.
[141] https://dvha.vermont.gov/sites/dvha/files/documents/providers/Pharmacy/VERMONT%20PDL_03.12.2021.pdf
[142] http://www.vtmedicaid.com/assets/advisories/January2019Advisory.pdf
[143] https://www.virginiamedicaidpharmacyservices.com/provider/external/medicaid/vamps/doc/en-us/VAmed-PDLquick-20210101.pdf
[144] https://www.virginiamedicaidpharmacyservices.com/provider/preferred-drug-list

© 2023 Christine PJ et al. *JAMA Health Forum.*

Washington[145]	Excluded	Yes	January 2018	PA repealed for preferred buprenorphine-naloxone formulations, though still required for non-preferred formulations.	146,147,148
West Virginia	No	No		PA for buprenorphine-naloxone formulations still in place.	149,150,151, 152
Wisconsin	Excluded	Yes	July 2018	PA repealed for preferred buprenorphine-naloxone formulations, though still required for non-preferred formulations.	153,154,155
Wyoming	No	No		PA still required for all formulations of buprenorphine-naloxone.	156

[145] Washington amended their state law on July 28, 2019 to formalize the requirement that at least one form of MOUD must be provided without a PA. However, we find that Apple Health (the state's Medicaid fee for service plan) removed their PA for preferred brand-name buprenorphine-naloxone film formulations (Suboxone) in January 2018.
[146] https://www.hca.wa.gov/assets/billers-and-providers/Apple_Health_PDL_01012018.pdf
[147] https://www.hca.wa.gov/assets/billers-and-providers/ah-ffs-pdl-20180101.pdf
[148] https://lawfilesext.leg.wa.gov/biennium/2019-20/Pdf/Bills/Session%20Laws/Senate/5380-S.SL.pdf?cite=2019%20c%20314%20%C2%A7%2038
[149] https://dhhr.wv.gov/bms/BMS%20Pharmacy/Documents/Preferred%20Drug%20List/2021/WV%20PDL%202021.Q2c.pdf
[150] https://h2cstrategies.com/2020/09/22/its-time-for-wv-to-eliminate-prior-authorization-for-substance-use-disorder-services-and-medications/
[151] https://dhhr.wv.gov/bms/BMS%20Pharmacy/Documents/Drug%20PA%20Criteria/Suboxone%20Policy%20v2021.%201a.pdf
[152] https://dhhr.wv.gov/bms/BMS%20Pharmacy/Documents/Prior%20Authorizations%20Forms/Drug%20Specific%20PA%20forms/Suboxone%20PA%20Form% 2020153b%20v2.pdf
[153] https://www.forwardhealth.wi.gov/WIPortal/content/provider/medicaid/pharmacy/pdl/archived_pdllists.htm.spage
[154] https://www.dhs.wisconsin.gov/forms/f00081-0718.pdf
[155] https://www.dhs.wisconsin.gov/publications/p02294-06-19.pdf
[156] http://www.wymedicaid.org/sites/default/files/ghs-files/pdl/2021-02-22/pdl-effective-02-24-2021.pdf

© 2023 Christine PJ et al. *JAMA Health Forum.*

eFigure 2. Visual Depiction of Panel Data and Treatment Timing

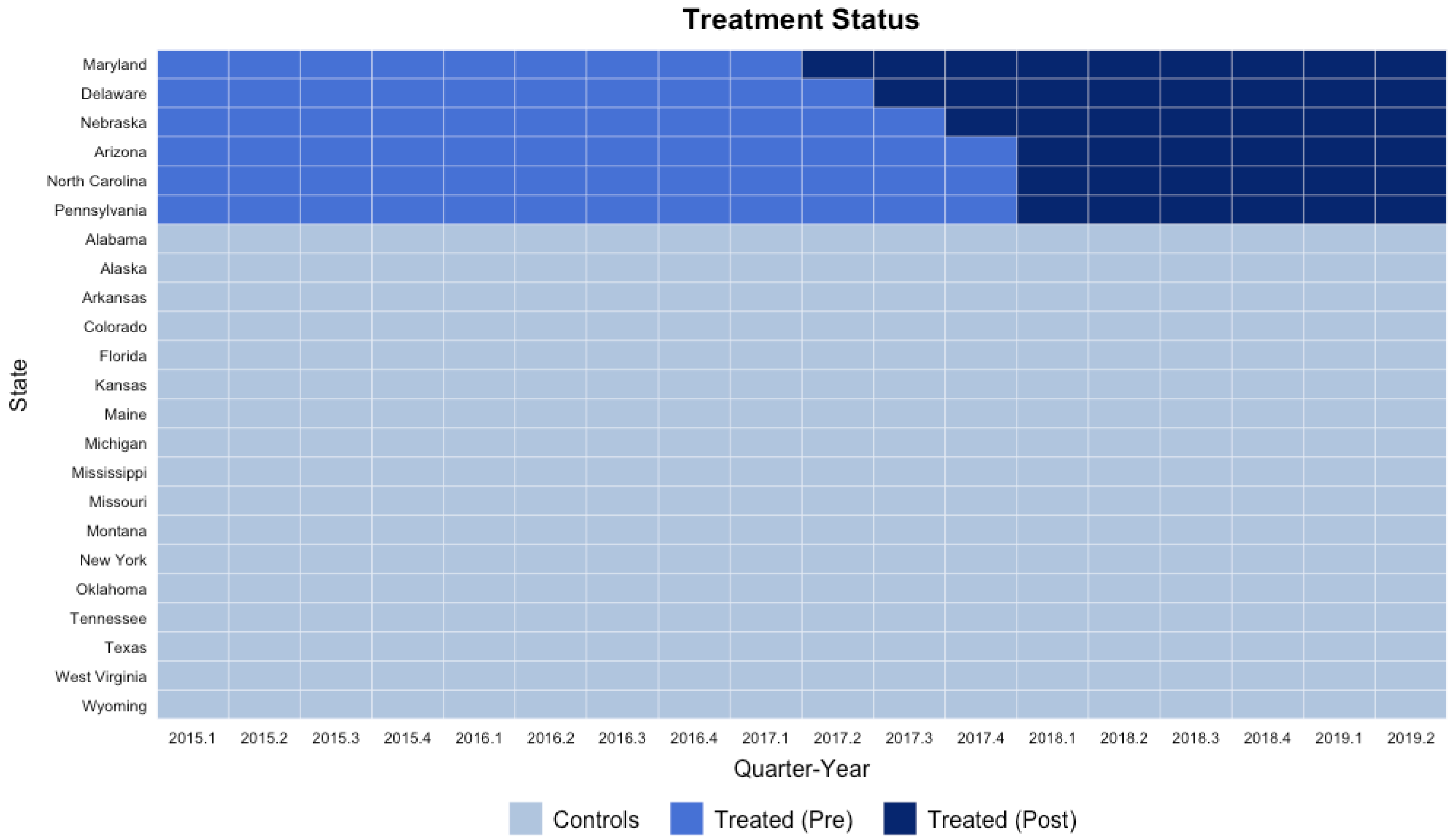

© 2023 Christine PJ et al. *JAMA Health Forum.*

eFigure 3. Unadjusted Log Buprenorphine Prescriptions Over Time by State and by Prior Authorization Policy Status, 2015-2019

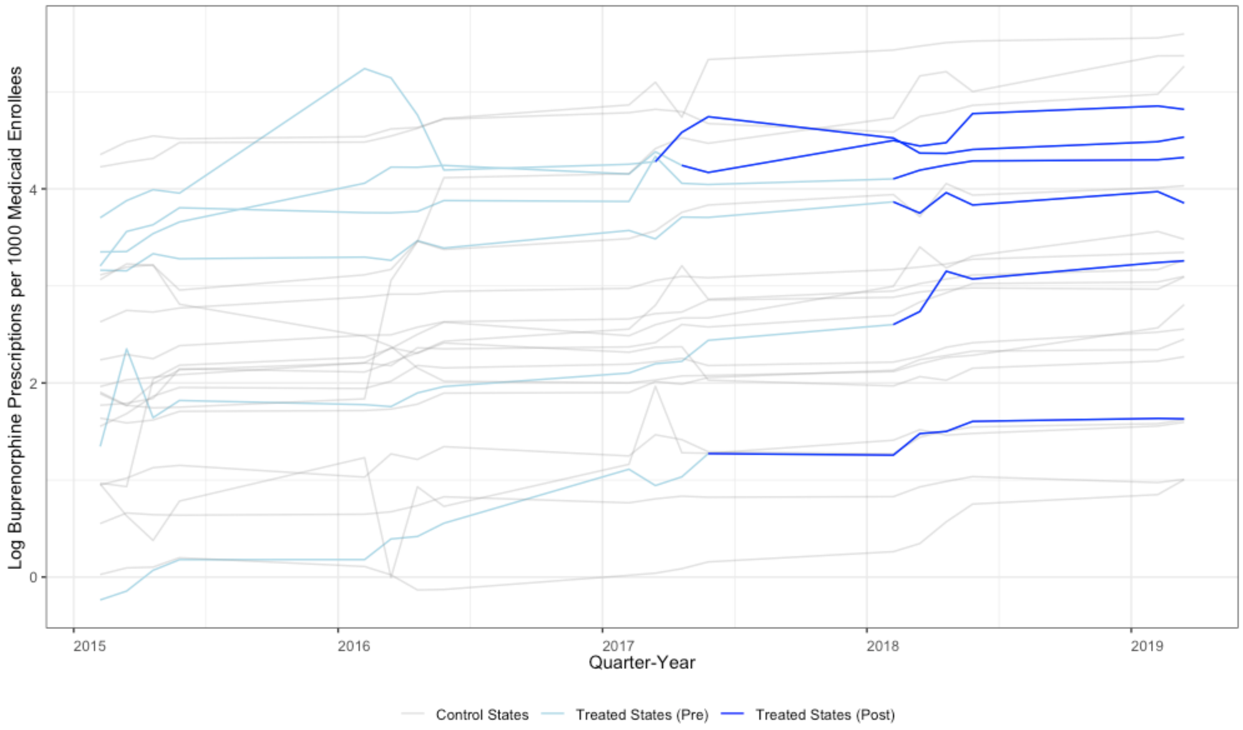

© 2023 Christine PJ et al. *JAMA Health Forum.*

eFigure 4. Visual Depiction of Panel Data and Treatment Timing for Expanded Sample of 40 States Where Medicaid Fee-for-Service (FFS) PA Policies for Buprenorphine Are Not Required to Be the Same

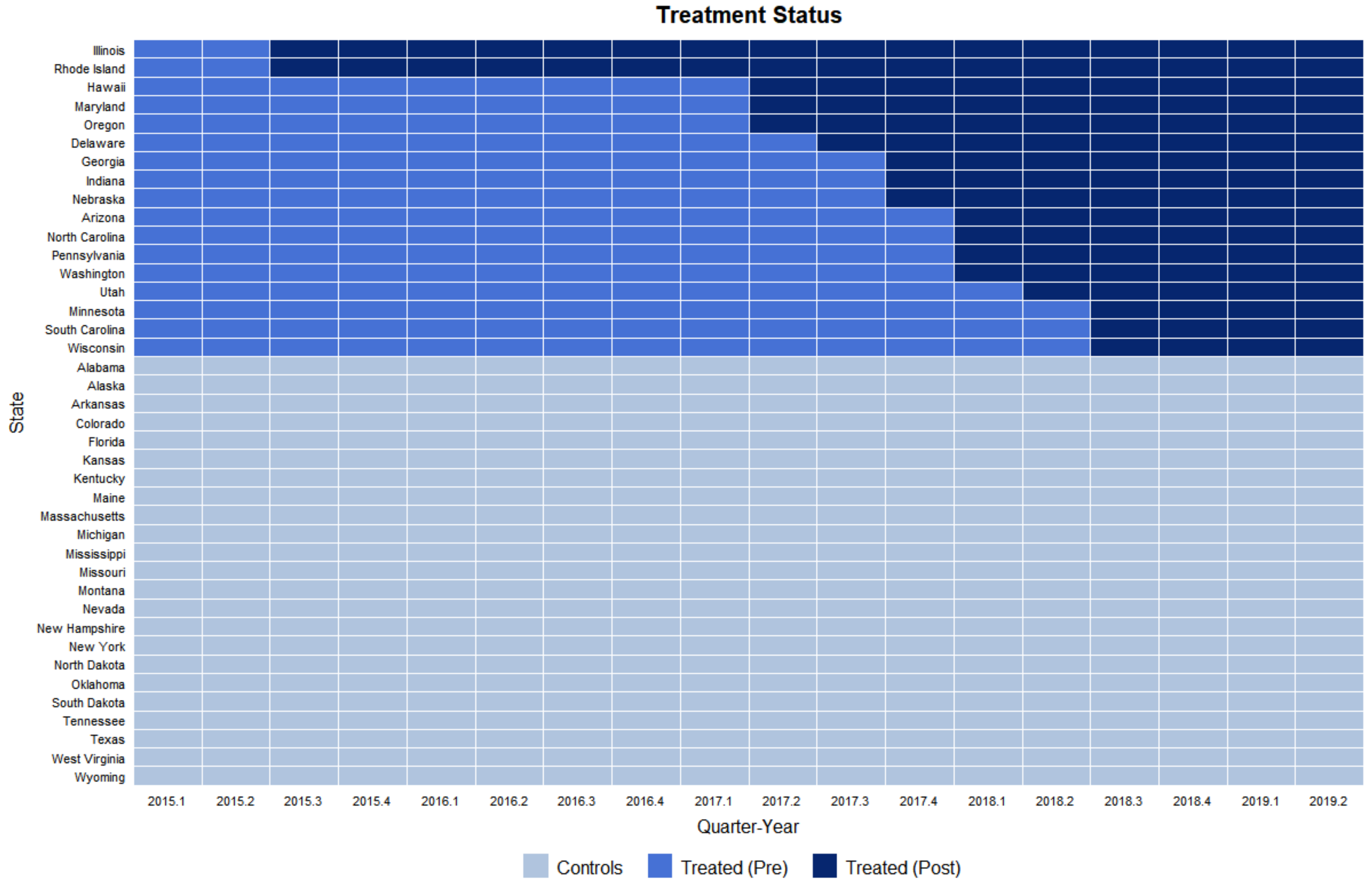

© 2023 Christine PJ et al. *JAMA Health Forum.*

eFigure 5. Event Study Estimates of Percent Change in Buprenorphine Prescriptions per 1000 Medicaid Enrollees Comparing States That Repealed PAs vs Those That Maintained PAs in Sensitivity Analysis Restricted to States Where Medicaid FFS PA Policies for Buprenorphine Are Known to Apply to All Medicaid Enrollees and Including Illinois

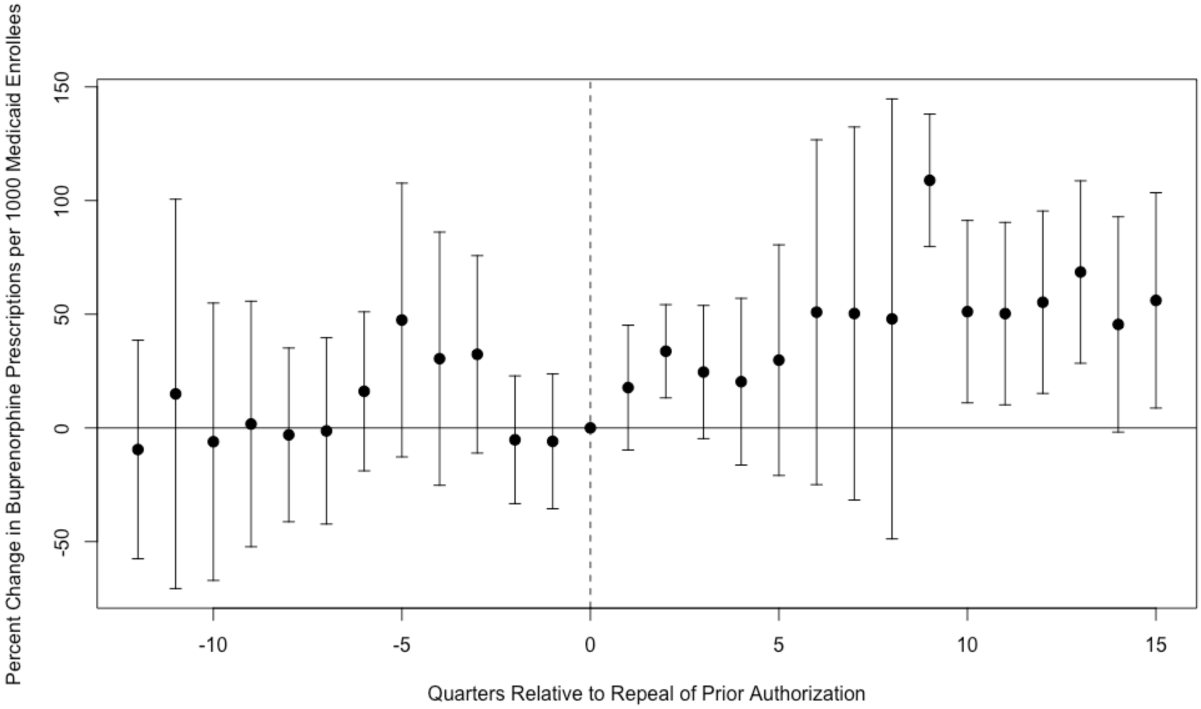

[a] Same sample as main analyses in paper but also includes Illinois which is the only state contributing to the effect estimates after the 9th quarter of the follow-up period (n=24 states, 432 state-quarters).

© 2023 Christine PJ et al. *JAMA Health Forum.*

eFigure 6. Event Study Estimates of Percent Change in Buprenorphine Prescriptions per 1000 Medicaid Enrollees Comparing States That Repealed PAs vs Those That Maintained PAs in sensitivity Analysis With Expanded Sample Not Restricted to States Where Medicaid FFS PA Policies for Buprenorphine Are Known to Apply to All Medicaid Enrollees

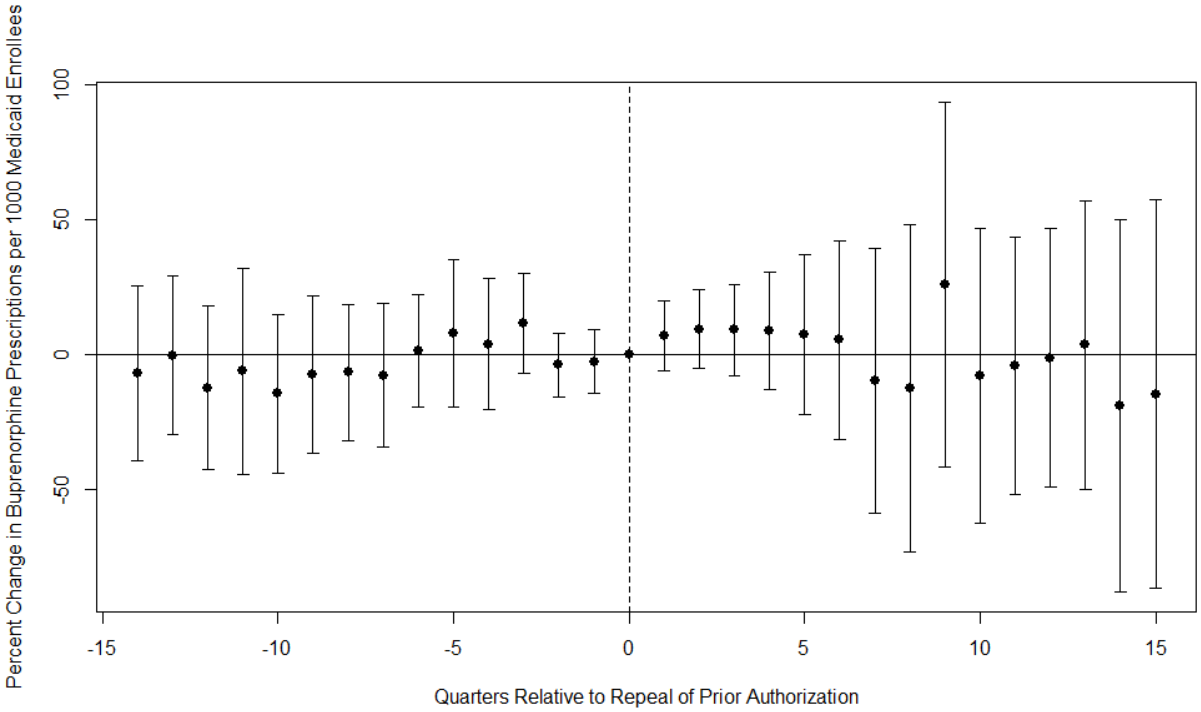

[a] Sample includes states where Medicaid FFS and MCO prior authorization policies are known to apply to all Medicaid enrollees, as well as states where prior authorization policies are allowed to differ (n=40 states, 720 state-quarters).

© 2023 Christine PJ et al. *JAMA Health Forum.*

eFigure 7. Decomposition of 2 x 2 Difference-in-Differences Estimates and Weights

Panel A: Difference-in-differences estimates for prior authorization removal in model with no covariates

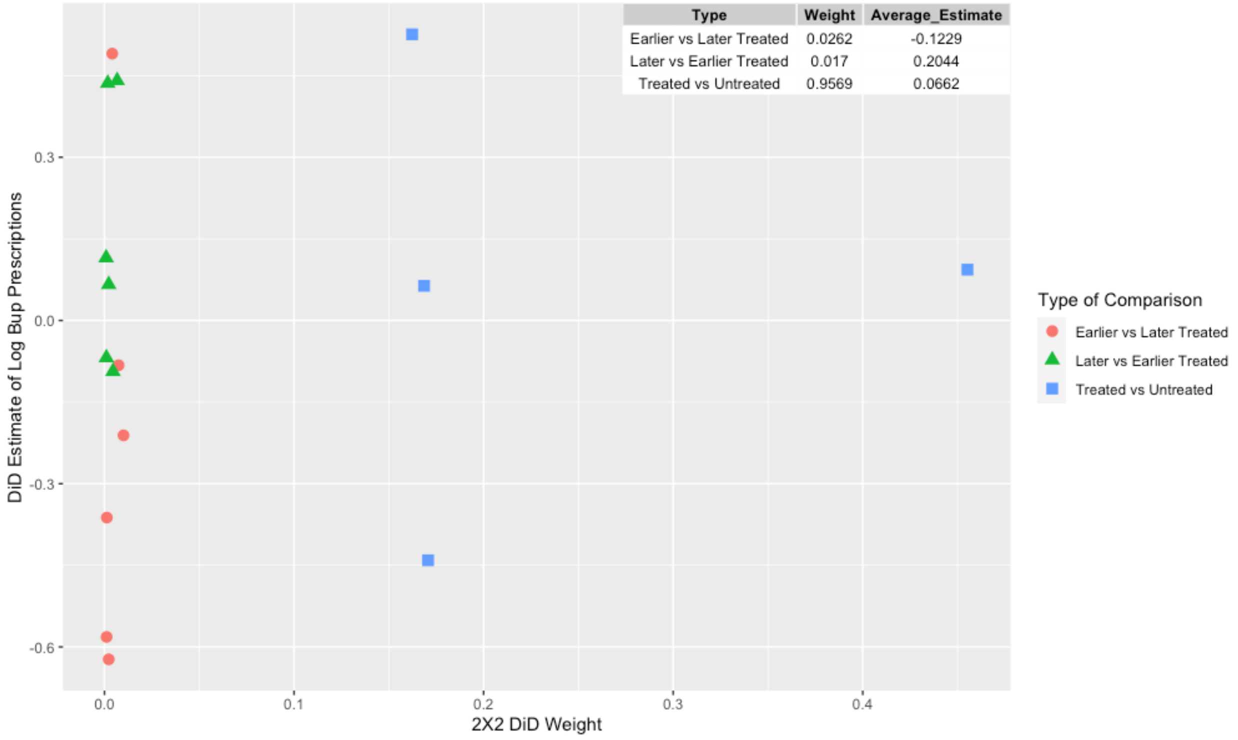

Panel B: Difference-in-differences estimates for prior authorization removal in full model with covariates

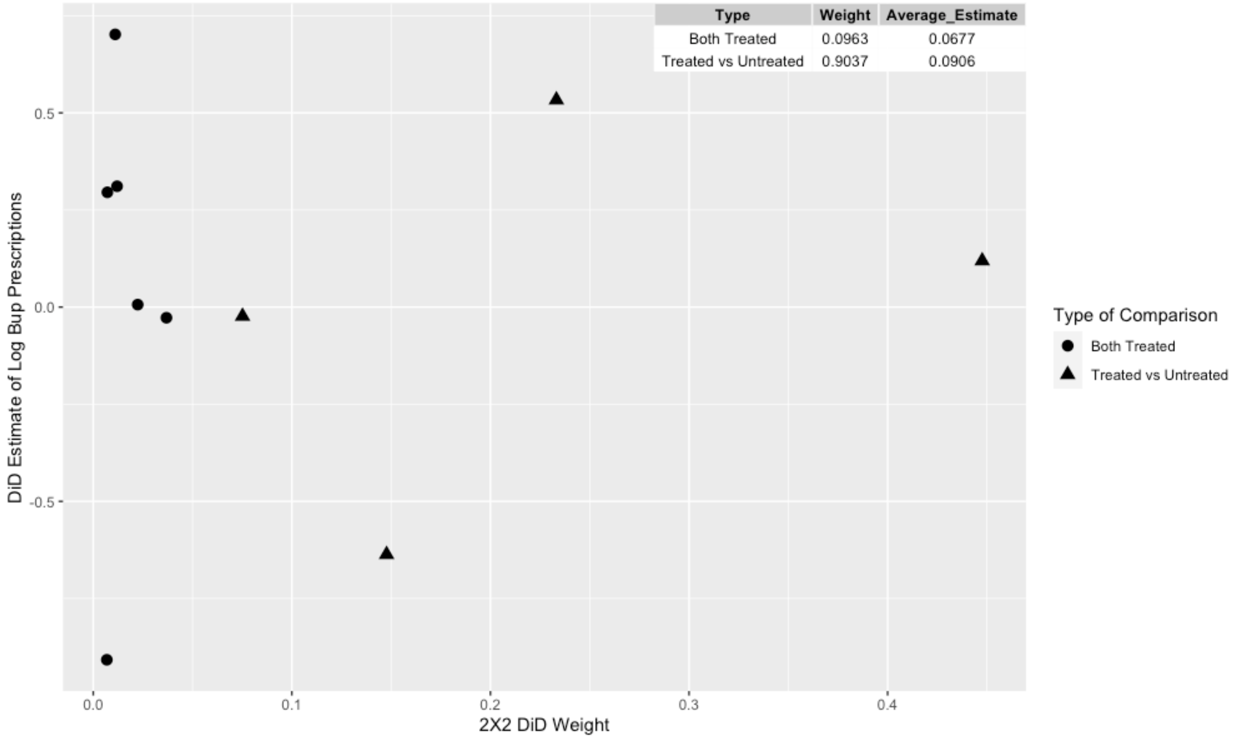

© 2023 Christine PJ et al. *JAMA Health Forum.*

eFigure 8. Sensitivity Analyses for the Effect of Prior Authorization Removal on Mean Difference in Log Buprenorphine Prescriptions per 1000 Medicaid Enrollees Using Different Estimators to Account for Variation in Treatment Timing[a]

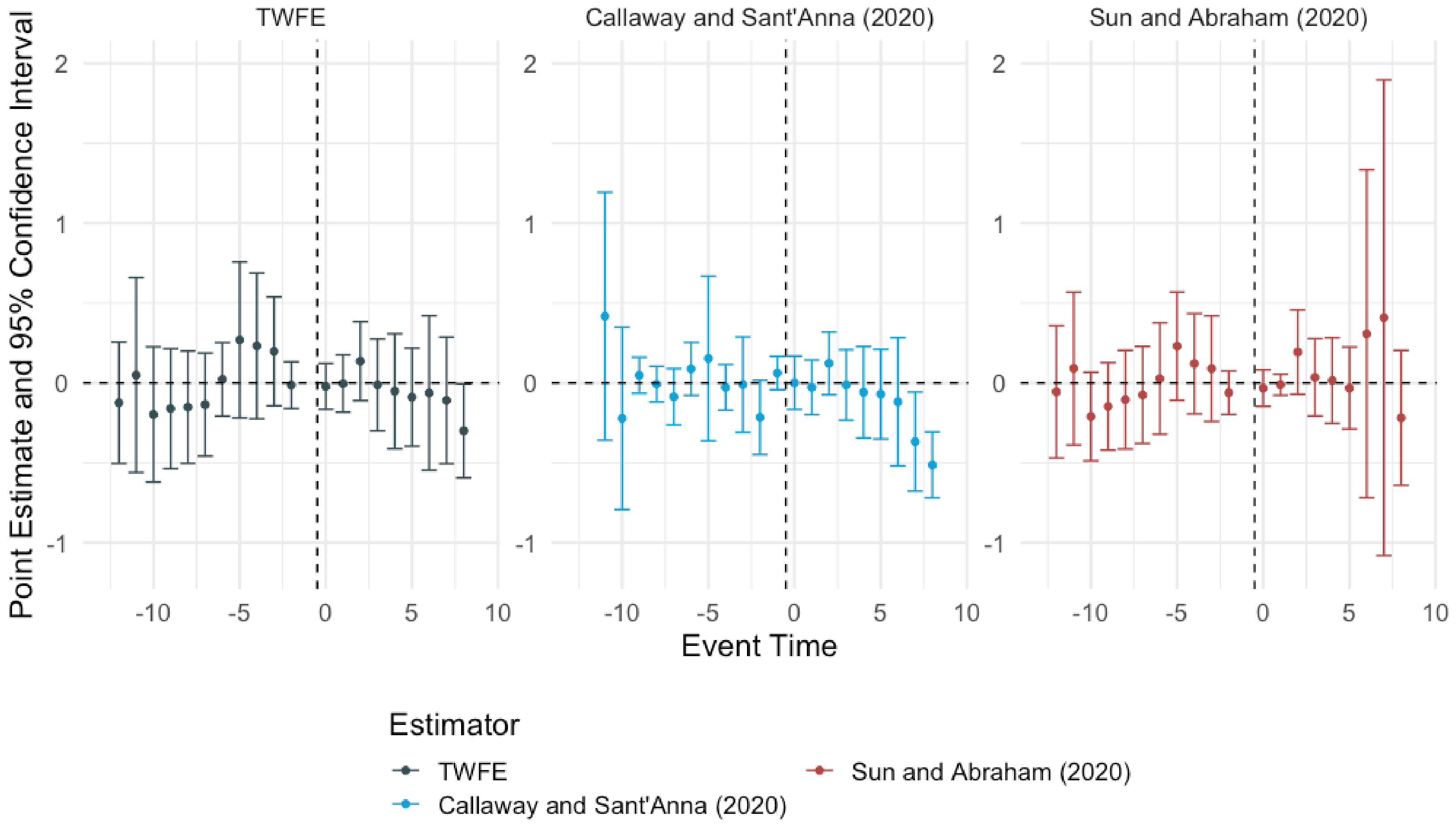

Abbreviations: TWFE = two-way fixed effects

[a] Models use the log of buprenorphine prescriptions as the outcome of interest. Event time refers to the number of quarters relative to prior authorization removal. TWFE and Sun and Abraham models control for all covariates in the main model of the paper. Callaway and Sant'Anna model controls only for opioid overdose deaths per 100,000 residents and Medicaid expansion status as additional covariate inclusion resulted in more extreme inverse probability of treatment weights. Model results are fairly similar across the estimators, with no significant pre-period non-parallel trends.

© 2023 Christine PJ et al. *JAMA Health Forum.*

eFigure 9. Generalized Synthetic Control Sensitivity Analyses for the Effect of Prior Authorization Removal on the Mean Difference in Log Buprenorphine Prescriptions per 1000 Medicaid Enrollees[a]

Panel A: Mean difference in log buprenorphine prescriptions (dark line) and 95% confidence intervals (shaded area)

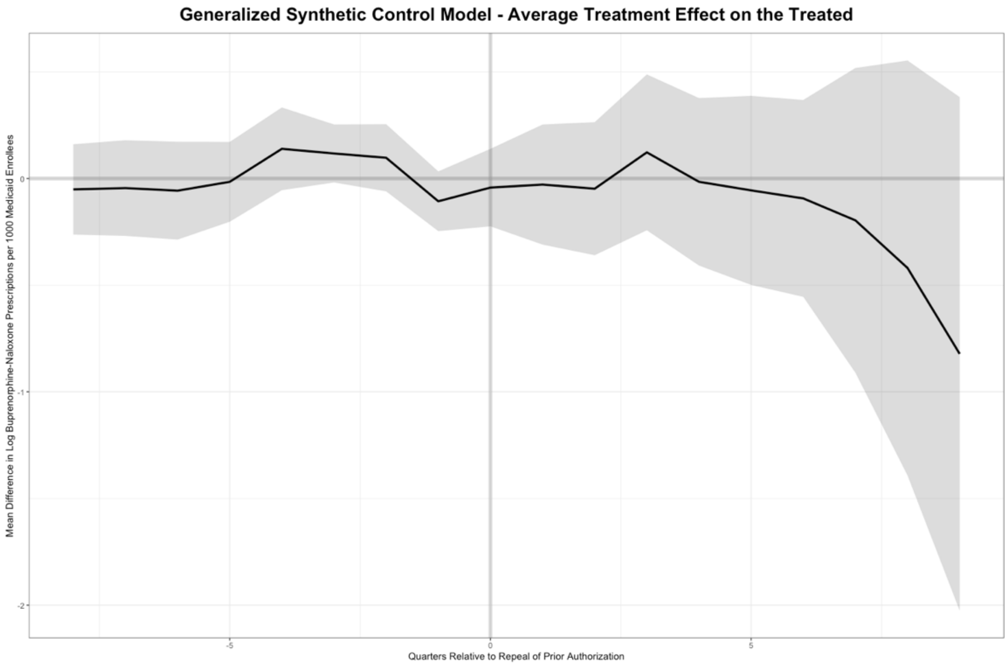

Panel B: Mean log buprenorphine prescriptions comparing average of treated states (black solid line) with counterfactual synthetic control average (blue dashed line)

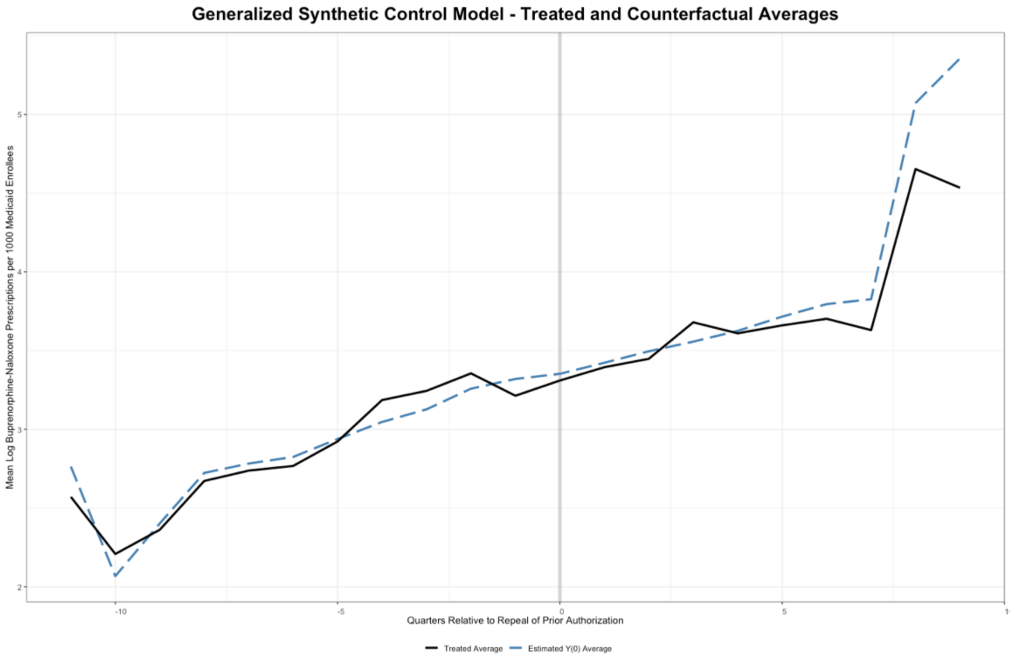

[a] Models include the same covariates as the main difference-in-differences models:

© 2023 Christine PJ et al. *JAMA Health Forum.*

eTable 2. Summary of Sensitivity Analyses and Point Estimates for the Effect of Prior Authorization Removal on Buprenorphine Prescriptions per 1000 Medicaid Enrollees Using Different Methods

Model	Mean difference in prescriptions on log scale (95% CI)	Percent change in prescriptions (95% CI)	Interpretation
Two-way fixed effects	-0.014 (-0.395, 0.367)	-1.4 (-31.2, 41.4)	Average treatment effect on treated
Sun and Abraham (2020)	0.150 (-0.174, 0.475)	16.2 (-16.0, 60.8)	Average treatment effect on treated using last-to-be-treated states as controls
Callaway and Sant'Anna (2021)[a]	-0.115 (-0.307, 0.077)	-10.9 (-25.8, 7.1)	Average treatment effect on treated based on event-study/dynamic aggregation using not-yet-treated states as controls
Generalized Synthetic Control	-0.070 (-0.462, 0.322)	-6.8 (-37.0, 37.9)	Average treatment effect on the treated, averaged over all time periods

[a] Callaway and Sant'Anna model only controls for opioid overdose deaths per 100,000 residents and Medicaid expansion status as noted above.

© 2023 Christine PJ et al. *JAMA Health Forum.*

eTable 3. Sensitivity Analyses for the Effect of Prior Authorization Removal on Buprenorphine Prescriptions Using Different Exposure Lag Periods[a]

Model	Mean difference in prescriptions on log scale (95% CI)	Percent change in prescriptions (95% CI)
Main model with no PA removal lag	-0.014 (-0.395, 0.367)	-1.4 (-31.2, 41.4)
Model with 6-month PA removal lag	0.026 (-0.350, 0.403)	2.7 (-28.1, 46.5.0)
Model with 12-month PA removal lag	-0.067 (-0.366, 0.313)	-6.5 (-34.7, 34.0)

[a] Difference-in-difference estimates from two-way fixed effects models. All models control state fixed effects, year fixed effects, and the following state-level covariates: opioid overdose deaths per 100,000, log number of X-waivered buprenorphine providers, number of individuals on methadone through an opioid treatment program per 100,000, percent poverty, Medicaid expansion status, proportion of the state's Medicaid enrollees covered under a comprehensive managed care plan, and whether or not a state mandates use of a prescription drug monitoring program. Standard errors are clustered at the state level.

© 2023 Christine PJ et al. *JAMA Health Forum.*

eTable 4. Sensitivity Analyses for the Effect of Prior Authorization Removal on Buprenorphine Prescriptions per 1000 Medicaid Enrollees Using Different Study Samples and Prior Authorization Categorizations[a]

Model	Mean difference in prescriptions on log scale (95% CI)	Percent change in prescriptions (95% CI)
Main model	-0.014 (-0.395, 0.367)	-1.4 (-31.2, 41.4)
Alaska categorized as PA removed[b]	-0.002 (-0.321, 0.317)	-0.2 (-25.1, 34.9)
Florida categorized as PA removed[c]	-0.015 (-0.360, 0.330)	-1.5 (-28.9, 36.5)
Include Vermont[d]	0.019 (-0.313, 0.351)	1.9 (-25.6, 39.6)

[a] Difference-in-difference estimates from two-way fixed effects models. All models control state fixed effects, year fixed effects, and the following state-level covariates: opioid overdose deaths per 100,000, log number of X-waivered buprenorphine providers, number of individuals on methadone through an opioid treatment program per 100,000, percent poverty, Medicaid expansion status, proportion of the state's Medicaid enrollees covered under a comprehensive managed care plan, and whether or not a state mandates use of a prescription drug monitoring program. Standard errors are clustered at the state level.
[b] Main models treat Alaska as a control state (never removed prior authorization). As explained in eTable 1, Alaska removed their prior authorization for the first 28-day fill of buprenorphine-naloxone in April 2017. This sensitivity analysis treats Alaska as removing their prior authorization in April 2017.
[c] Main models treat Florida as control state (never removed prior authorization). As explained in eTable 1, Florida removed their prior authorization for the first 7-day fill of buprenorphine-naloxone in February 2018. This sensitivity analysis treats Florida as removing their prior authorization in February 2018.
[d] Main models exclude Vermont which had very high use of buprenorphine per 1000 Medicaid enrollees, more than double the next closest state. This sensitivity analysis keeps Vermont in the sample.

© 2023 Christine PJ et al. *JAMA Health Forum.*

eTable 5. National Drug Codes Used for Buprenorphine Prescriptions in Medicaid State Drug Utilization Database[a]

Proprietary Name, if listed	NDC Package Code
Bunavail	59385-012-30
Bunavail	59385-014-30
Bunavail	59385-016-30
Buprenorphine	0228-3153-03
Buprenorphine	42858-501-03
Buprenorphine	42858-502-03
Buprenorphine	0228-3156-03
Buprenorphine	62756-459-64
Buprenorphine	62756-459-83
Buprenorphine	62756-460-64
Buprenorphine	62756-460-83
Buprenorphine	61786-911-02
Buprenorphine	61786-912-02
Buprenorphine	71335-0353-1
Buprenorphine	71335-0353-2
Buprenorphine	71335-0353-3
Buprenorphine	71335-0353-4
Buprenorphine	71335-0353-5
Buprenorphine	71335-0353-6
Buprenorphine	71335-0353-7
Buprenorphine	70518-0711-0
Buprenorphine	70518-0711-1
Buprenorphine	67046-992-30
Buprenorphine	67046-993-30
Buprenorphine	67046-994-30
Buprenorphine	67046-995-30
Buprenorphine	0093-5378-56
Buprenorphine	0093-5379-56
Buprenorphine	71335-0950-2
Buprenorphine	71335-0950-3
Buprenorphine	71335-0950-6
Buprenorphine	58118-3156-8
Buprenorphine	71335-0950-5
Buprenorphine	70518-1557-0
Buprenorphine	70518-1625-0
Buprenorphine	71335-0950-4
Buprenorphine	71335-0950-7
Buprenorphine	71335-0950-1

© 2023 Christine PJ et al. *JAMA Health Forum.*

Buprenorphine	71335-1154-2
Buprenorphine	71335-1154-3
Buprenorphine	71335-1154-9
Buprenorphine	71335-1154-6
Buprenorphine	71335-1154-1
Buprenorphine	71335-1154-5
Buprenorphine	71335-1154-7
Buprenorphine	71335-1154-4
Buprenorphine	71335-1154-8
Buprenorphine	70518-2218-0
Buprenorphine	70518-2217-0
Buprenorphine	60687-492-21
Buprenorphine	60687-481-21
Buprenorphine	70518-0711-2
Buprenorphine (E)	50090-2924-0
Buprenorphine and Naloxone	50383-287-93
Buprenorphine and Naloxone	50383-294-93
Buprenorphine and Naloxone	0228-3154-73
Buprenorphine and Naloxone	0228-3155-73
Buprenorphine and Naloxone	62175-452-32
Buprenorphine and Naloxone	62175-458-32
Buprenorphine and Naloxone	62756-969-64
Buprenorphine and Naloxone	62756-969-83
Buprenorphine and Naloxone	60429-586-30
Buprenorphine and Naloxone	60429-586-33
Buprenorphine and Naloxone	60429-587-30
Buprenorphine and Naloxone	60429-587-33
Buprenorphine and Naloxone	60846-970-03
Buprenorphine and Naloxone	60846-971-03
Buprenorphine and Naloxone	62756-970-64
Buprenorphine and Naloxone	62756-970-83
Buprenorphine and Naloxone	63629-7270-1
Buprenorphine and Naloxone	63629-7270-2
Buprenorphine and Naloxone	53217-138-30
Buprenorphine and Naloxone	0093-5720-56
Buprenorphine and Naloxone	0093-5721-56
Buprenorphine and Naloxone	43598-581-30
Buprenorphine and Naloxone	43598-579-30
Buprenorphine and Naloxone	0378-8768-93
Buprenorphine and Naloxone	0378-8767-93
Buprenorphine and Naloxone	43598-582-30

© 2023 Christine PJ et al. *JAMA Health Forum.*

Buprenorphine and Naloxone	43598-580-30
Buprenorphine and Naloxone	47781-356-03
Buprenorphine and Naloxone	47781-357-03
Buprenorphine and Naloxone	47781-355-03
Buprenorphine and Naloxone	47781-358-03
Buprenorphine and Naloxone	0781-7249-64
Buprenorphine and Naloxone	0781-7227-64
Buprenorphine and Naloxone	0781-7238-64
Buprenorphine and Naloxone	0781-7216-64
Buprenorphine and Naloxone	0093-2155-33
Buprenorphine and Naloxone	0378-8767-16
Buprenorphine and Naloxone	52427-692-03
Buprenorphine and Naloxone	52427-694-03
Buprenorphine and Naloxone	52427-698-03
Buprenorphine and Naloxone	52427-712-03
Buprenorphine and Naloxone	43598-580-01
Buprenorphine and Naloxone	43598-580-30
Buprenorphine and Naloxone	43598-581-01
Buprenorphine and Naloxone	43598-581-30
Buprenorphine and Naloxone	70518-2311-0
Buprenorphine and Naloxone	70518-2327-0
Buprenorphine and Naloxone	71335-1378-1
Buprenorphine and Naloxone	0228-3154-03
Buprenorphine and Naloxone	0228-3155-03
Buprenorphine and Naloxone	71335-1296-1
Buprenorphine and Naloxone	71335-1296-2
Buprenorphine and Naloxone	42858-601-03
Buprenorphine and Naloxone	42858-602-03
Buprenorphine and Naloxone	0378-8765-93
Buprenorphine and Naloxone	0378-8766-93
Buprenorphine HCl	43063-753-06
Buprenorphine HCl	0054-0176-13
Buprenorphine HCl	0054-0177-13
Buprenorphine HCl	55700-302-30
Buprenorphine HCl	55700-303-30
Buprenorphine HCl	67046-998-30
Buprenorphine HCl	67046-999-30
Buprenorphine HCl	76519-1170-3
Buprenorphine HCl	76519-1170-1
Buprenorphine HCl	58118-0176-8
Buprenorphine HCl	58118-0177-8

© 2023 Christine PJ et al. *JAMA Health Forum.*

Buprenorphine HCl	76519-1170-5
Buprenorphine HCl	76519-1170-2
Buprenorphine HCl	76519-1170-4
Buprenorphine HCl	76519-1170-0
Buprenorphine HCl	71335-1163-1
Buprenorphine HCl	71335-1163-4
Buprenorphine HCl	71335-1163-3
Buprenorphine HCl	71335-1163-5
Buprenorphine HCl	71335-1163-2
Buprenorphine HCl	71335-1163-7
Buprenorphine HCl	71335-1163-9
Buprenorphine HCl	71335-1163-6
Buprenorphine HCl	71335-1163-8
Buprenorphine HCl	70518-2014-0
Buprenorphine HCl	70518-2226-0
Buprenorphine HCl and Naloxone HCl	0406-1923-03
Buprenorphine HCl and Naloxone HCl	0406-1923-09
Buprenorphine HCl and Naloxone HCl	0406-1924-03
Buprenorphine HCl and Naloxone HCl	0406-1924-09
Buprenorphine HCl and Naloxone HCl	70518-1007-0
Buprenorphine HCl and Naloxone HCl	50268-144-15
Buprenorphine HCl and Naloxone HCl	50268-145-15
Buprenorphine HCl and Naloxone HCl	42291-174-30
Buprenorphine HCl and Naloxone HCl	42291-175-30
Buprenorphine HCl and Naloxone HCl	65162-415-03
Buprenorphine HCl and Naloxone HCl	65162-415-09
Buprenorphine HCl and Naloxone HCl	65162-416-03
Buprenorphine HCl and Naloxone HCl	65162-416-09
Buprenorphine HCl and Naloxone HCl	70518-1684-0
Buprenorphine HCl and Naloxone HCl	71335-1514-1
Buprenorphine HCl and Naloxone HCl	71335-1514-2
Buprenorphine HCl and Naloxone HCl (U)	54569-6408-0
Buprenorphine HCl and Naloxone HCl (U)	63629-5074-1
Buprenorphine HCl	49349-554-02
Buprenorphine HCl	55154-4962-0
Buprenorphine HCl	55154-4962-4
Buprenorphine HCl	55154-4962-6
Buprenorphine HCl	64725-0930-3
Buprenorphine HCl	64725-0930-4
buprenorphine hydrochloride	43063-667-06
buprenorphine hydrochloride	50383-924-93

© 2023 Christine PJ et al. *JAMA Health Forum.*

buprenorphine hydrochloride	50383-930-93
buprenorphine hydrochloride	63629-7125-1
buprenorphine hydrochloride	63629-7125-2
buprenorphine hydrochloride	63629-7125-3
buprenorphine hydrochloride	63629-7125-4
buprenorphine hydrochloride	63629-7125-5
buprenorphine hydrochloride	63629-7125-6
buprenorphine hydrochloride	63629-7125-7
buprenorphine hydrochloride	63629-7126-1
buprenorphine hydrochloride	63629-7126-2
buprenorphine hydrochloride	63629-7126-3
buprenorphine hydrochloride	63629-7126-4
buprenorphine hydrochloride	63629-7126-5
buprenorphine hydrochloride	63629-7126-9
buprenorphine hydrochloride	52125-678-02
buprenorphine hydrochloride	63629-7126-6
buprenorphine hydrochloride	63629-7126-7
buprenorphine hydrochloride	63629-7126-8
buprenorphine hydrochloride	70518-0442-0
buprenorphine hydrochloride	67046-990-30
buprenorphine hydrochloride	67046-991-30
buprenorphine hydrochloride	53217-246-30
buprenorphine hydrochloride	35356-556-30
buprenorphine hydrochloride	70518-2216-0
buprenorphine hydrochloride (E)	50090-1571-0
buprenorphine hydrochloride (U)	54569-6578-0
buprenorphine hydrochloride (U)	64725-1924-3
buprenorphine hydrochloride (U)	64725-1924-4
Buprenorphine hydrochloride (U)	63629-4092-1
Buprenorphine hydrochloride (U)	63629-4092-2
buprenorphine hydrochloride and naloxone hydrochloride dihydrate	0054-0188-13
buprenorphine hydrochloride and naloxone hydrochloride dihydrate	0054-0189-13
buprenorphine hydrochloride and naloxone hydrochloride dihydrate	0406-8005-03
buprenorphine hydrochloride and naloxone hydrochloride dihydrate	0406-8020-03
buprenorphine hydrochloride and naloxone hydrochloride dihydrate	69189-0591-1
buprenorphine hydrochloride and naloxone hydrochloride dihydrate	71335-1720-1
buprenorphine hydrochloride and naloxone hydrochloride dihydrate	71335-1720-2

© 2023 Christine PJ et al. *JAMA Health Forum.*

buprenorphine hydrochloride and naloxone hydrochloride dihydrate	71335-1725-1
buprenorphine hydrochloride and naloxone hydrochloride dihydrate	71335-1653-1
Buprenorphine Hydrochloride Sublingual	0378-0923-93
Buprenorphine Hydrochloride Sublingual	0378-0924-93
Buprenorphine Hydrochloride Sublingual	70518-0652-0
Buprenorphine Hydrochloride Sublingual	70518-0652-1
Buprenorphine Hydrochloride Sublingual	67046-996-30
Buprenorphine Hydrochloride Sublingual	67046-997-30
Buprenorphine Hydrochloride Sublingual	70518-0652-2
Buprenorphine Hydrochloride-Naloxone Hydrochloride	00228-3154-09
Buprenorphine Hydrochloride-Naloxone Hydrochloride	00228-3155-09
Probuphine	58284-100-14
Probuphine	52440-100-14
Sublocade	12496-0100-1
Sublocade	12496-0300-1
Sublocade	12496-0100-2
Sublocade	12496-0100-5
Sublocade	12496-0300-2
Sublocade	12496-0300-5
Suboxone	53217-328-01
Suboxone	55700-147-30
Suboxone	12496-1202-3
Suboxone	12496-1204-3
Suboxone	12496-1208-3
Suboxone	12496-1212-3
Suboxone	55887-0312-04
Suboxone	55887-0312-15
Suboxone	66336-0015-30
Suboxone (U)	54569-6399-0
Suboxone (U)	63629-4028-1
Suboxone (U)	63629-4028-2
suboxone (U)	63629-4034-1
suboxone (U)	63629-4034-2
Suboxone (U)	54868-5707-0
Suboxone (U)	54868-5707-1
Suboxone (U)	54868-5707-2
Suboxone (U)	54868-5707-3
Suboxone (U)	54868-5707-4
Suboxone (U)	35356-004-07

© 2023 Christine PJ et al. *JAMA Health Forum.*

Suboxone (U)	35356-004-30
Suboxone (U)	54868-5750-0
Suboxone (U)	63629-4034-3
Suboxone (U)	63629-4034-4
Suboxone	12496-1283-2
Suboxone	12496-1306-2
Subutex	12496-1310-2
Subutex	12496-1278-2
Subutex	63874-1174-03
Zubsolv	54123-114-30
Zubsolv	54123-907-30
Zubsolv	54123-914-30
Zubsolv	54123-929-30
Zubsolv	54123-957-30
Zubsolv	54123-986-30
	55700-0184-30
	59385-0012-01
	59385-0014-01
	59385-0016-01
	68308-0202-30
	68308-0208-30
	35356-0555-30
	68258-2991-03
	12496-1202-01
	12496-1204-01
	12496-1208-01
	12496-1212-01
	16590-0666-30
	43063-0184-07
	43063-0184-30
	49999-0395-07
	49999-0395-15
	49999-0395-30
	52959-0304-30
	52959-0749-30
	54569-5496-00
	54569-5739-00
	54569-5739-01
	54569-5739-02
	55045-3784-03
	63874-1084-03

© 2023 Christine PJ et al. *JAMA Health Forum.*

	63874-1085-03
	66336-0016-30
	68071-1380-03
	68071-1510-03
	68258-2999-03
	00490-0051-00
	00490-0051-30
	00490-0051-60
	00490-0051-90
	16590-0666-05
	16590-0667-05
	16590-0667-30
	16590-0667-90
	23490-9270-03
	23490-9270-06
	23490-9270-09
	49999-0638-30
	49999-0639-30
	63874-1173-03

[a] National Drug Codes (NDCs) provided by Lisa Clemans-Cope at Urban Institute. For more information regarding buprenorphine prescription data, see citations below.[2,3]

eReferences

1. Keshwani S, Maguire M, Goodin A, Lo-Ciganic WH, Wilson DL, Hincapie-Castillo JM. Buprenorphine Use Trends Following Removal of Prior Authorization Policies for the Treatment of Opioid Use Disorder in 2 State Medicaid Programs. *JAMA Health Forum*. 2022;3(6):e221757. doi:10.1001/jamahealthforum.2022.1757

2. Clemans-Cope L, Epstein M, Winiski E, Lynch V. Tracking Medicaid-Covered Prescriptions to Treat Opioid Use Disorder: Methodology Appendix. *Urban Institute*. Published online November 19, 2019:12.

3. Urban Institute. Tracking Medicaid-Covered Prescriptions to Treat Opioid Use Disorder. Published online August 26, 2020. Accessed December 1, 2019. https://www.urban.org/policy-centers/health-policy-center/projects/tracking-medicaid-covered-prescriptions-treat-opioid-use-disorder

4. Centers for Disease Control and Prevention. CDC Wonder Multiple Cause of Death. Accessed August 17, 2021. https://wonder.cdc.gov/

5. Substance Abuse and Mental Health Services Administration. National Survey of Substance Abuse Treatment Services (N-SSATS). Accessed February 25, 2022. https://www.samhsa.gov/data/data-we-collect/n-ssats-national-survey-substance-abuse-treatment-services

6. United States Census Bureau. Small Area Income and Poverty Estimates (SAIPE) Program. Accessed February 24, 2022. https://www.census.gov/programs-surveys/saipe.html

7. Kaiser Family Foundation. *Share of Medicaid Population Covered under Different Delivery Systems*. Accessed April 18, 2021. https://www.kff.org/medicaid/state-indicator/share-of-medicaid-population-covered-under-different-delivery-systems/?currentTimeframe=0&sortModel=%7B%22colId%22:%22Location%22,%22sort%22:%22asc%22%7D

8. Kaiser Family Foundation. *Status of State Action on the Medicaid Expansion Decision*. Accessed April 27, 2021. https://www.kff.org/health-reform/state-indicator/state-activity-around-expanding-medicaid-under-the-affordable-care-act/?currentTimeframe=0&sortModel=%7B%22colId%22:%22Location%22,%22sort%22:%22asc%22%7D

9. Lee B, Zhao W, Yang KC, Ahn YY, Perry BL. Systematic Evaluation of State Policy Interventions Targeting the US Opioid Epidemic, 2007-2018. *JAMA Netw Open*. 2021;4(2):e2036687. doi:10.1001/jamanetworkopen.2020.36687

10. *Medicaid Drug Utilization Review Annual Report*. Accessed February 14, 2023. https://www.medicaid.gov/medicaid/prescription-drugs/drug-utilization-review/drug-utilization-review-annual-report/index.html

© 2023 Christine PJ et al. *JAMA Health Forum.*

11. Roth J, Sant'Anna PHC, Bilinski A, Poe J. What's Trending in Difference-in-Differences? A Synthesis of the Recent Econometrics Literature. *arXiv e-prints*. Published online January 2022:arXiv:2201.01194.

12. Goodman-Bacon A. Difference-in-differences with variation in treatment timing. *Journal of Econometrics*. 2021;225(2):254-277. doi:10.1016/j.jeconom.2021.03.014

13. Callaway B, Sant'Anna PHC. Difference-in-Differences with multiple time periods. *Journal of Econometrics*. 2021;225(2):200-230. doi:10.1016/j.jeconom.2020.12.001

14. Sun L, Abraham S. Estimating dynamic treatment effects in event studies with heterogeneous treatment effects. *Journal of Econometrics*. 2021;225(2):175-199. doi:10.1016/j.jeconom.2020.09.006

15. Cunningham S. *Causal Inference*. Yale University Press; 2021. doi:10.2307/j.ctv1c29t27

16. Bonander C, Humphreys D, Degli Esposti M. Synthetic Control Methods for the Evaluation of Single-Unit Interventions in Epidemiology: A Tutorial. *Am J Epidemiol*. 2021;190(12):2700-2711. doi:10.1093/aje/kwab211

17. Xu Y. Generalized Synthetic Control Method: Causal Inference with Interactive Fixed Effects Models. *Political Analysis*. 2017;25(1):57-76. doi:10.1017/pan.2016.2
