# Supplementary material for: Removal of Medicaid Prior Authorization Requirements and Buprenorphine Treatment for Opioid Use Disorder
Source: JAMA Health Forum. 2023 Oct 20;4(10):e233549. doi: 10.1001/jamahealthforum.2023.3549 (PMC10589810; doi:10.1001/jamahealthforum.2023.3549)
Supplement: Supplement 2. — Data Sharing Statement [file jamahealthforum-e233549-s002.pdf]

Data Sharing Statement

Christine. Removal of Medicaid Prior Authorization Requirements and Buprenorphine Treatment for Opioid Use Disorder. *JAMA Health Forum*. Published October 20, 2023. doi:10.1001/jamahealthforum.2023.3549

Data
Data available: Yes
Data types: Participant data with identifiers
How to access data: paul.christine@cuanschutz.edu
When available: With publication

Supporting Documents
Document types: Other (please specify)
Additional Information: Prior authorization policy data and sources, NDC codes for buprenorphine prescriptions
How to access documents: This will be provided in the online supplement to the publication for others to freely use and adapt
When available: With publication

Additional Information
Who can access the data: anyone requesting the data
Types of analyses: for any purpose
Mechanisms of data availability: via emailing the corresponding author
